# Machine Learning‐Guided Repositioning of a SARS‐CoV‐2‐Targeting Molecular Series as Cruzain Inhibitors

**DOI:** 10.1002/cmdc.202500630

**Published:** 2026-01-31

**Authors:** Rafael F. Lameiro, Luiz F. Barbosa, Evelin R. Cardoso, Beatriz Siqueira Ho, Felipe Cardoso Prado Martins, Bruna C. de Melo, Fabiana Rosini, Anwar Shamim, Priscila M. Souza, Wellington Falcão de Souza, Carlos A. Montanari

**Affiliations:** ^1^ Medicinal and Biological Chemistry Group São Carlos Institute of Chemistry University of São Paulo Avenida Trabalhador Sancarlense, 400 São Carlos SP 13566–590 Brazil

**Keywords:** calorimetry, chemoinformatics, cruzain, machine learning, medicinal chemistry

## Abstract

Drug repurposing and repositioning are concepts that involve identifying alternative therapeutic uses for existing drug candidates or molecular series. During the COVID‐19 pandemic, hundreds of antivirals were developed, many of which remain unexplored for other diseases. Concurrently, machine learning (ML) has become a valuable tool in early drug discovery for screening the most promising compounds for a target. In this work, an ExtraTrees ML model is developed to predict inhibitory activity against cruzain, the main cysteine protease of *Trypanosoma cruzi*, the causative agent of Chagas disease. The model is used to screen a proprietary library of peptidomimetic compounds originally designed to target SARS‐CoV‐2 M^pro^ and human cathepsin L. High‐affinity cruzain inhibitors are identified, some containing P1 moieties not previously reported in cruzain inhibitors, expanding the known chemical space for this target. Selected hits are validated using isothermal titration calorimetry and some compounds display more favorable enthalpic and entropic contributions to binding than similar peptidomimetic nitrile‐based inhibitors. Notably, this is achieved without highly lipophilic R‐groups, preserving drug‐like properties. This work also highlights how compound libraries derived from global health efforts can be effectively repurposed for neglected tropical diseases with ML models.

## Graphical abstract

An ExtraTrees machine learning (ML) model is used to reposition a SARS‐CoV‐2‐directed library of peptidomimetic compounds as cruzain inhibitors for Chagas disease. Potent binders with a novel P1 moiety (for cruzain) are identified and validated by calorimetry. This work demonstrates how pandemic‐era drug libraries and ML tools can accelerate discovery for neglected diseases.

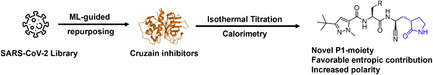

## 1 Introduction

“Repurposing” and “repositioning” in medicinal chemistry are two related terms that refer to using drugs or other bioactive compounds that were originally developed for a therapeutic use in a different context, for instance, to act on another target or disease.^[^ ^1^, ^2^, ^3^ ^]^ While often used interchangeably, we will define “repurposing” as finding new applications for approved drugs and “repositioning” as the identification of new targets and modes of action for compounds that are not approved drugs, that is, drug candidates and molecular series that did not reach the clinical phase.

Repositioning offers a way to rescue compounds that might have showed promise during development, but that were put aside on the preclinical phase or failed in clinical trials for their original indication. By leveraging existing chemical matter and structure–activity relationships, this approach offers advantages over traditional de novo drug discovery, such as reduced development timelines and lower costs. For molecular series in more advanced stages of the drug discovery pipeline, the compounds might also present improved ADME properties, acceptable solubility, and reduced toxicity.^[^ ^4^ ^,^ ^5^ ^]^ These attributes are of significant interest in neglected diseases research, where limited funding and resources often make it impractical to optimize entirely new chemical entities starting from early‐stage discovery.

Computational methods also play a powerful role in guiding repositioning by directing the chemical space toward compounds with a higher probability of presenting the desired activity on a target of interest. In particular, the increasing use of machine learning (ML) methods in drug discovery, where statistical methods and algorithms are used to identify patterns in compound property data, has significantly enhanced drug repurposing and repositioning efforts^[^ ^6^, ^7^, ^8^, ^9^ ^]^ and neglected diseases research.^[^ ^10^, ^11^, ^12^, ^13^ ^]^

Structural similarities between targets tend to be a good indication of a possible multitarget effect and, consequently, of a successful repositioning effort.^[^ ^14^ ^]^ A recent example is the development of nirmatrelvir, an inhibitor of the SARS‐CoV‐2 main protease (M^pro^), for which earlier hits were obtained by repurposing chemical series developed for the MERS‐CoV main protease.^[^ ^15^ ^]^ In fact, the COVID‐19 pandemic led to a widespread search for a cure, with efforts dedicated to repositioning^[^ ^16^ ^]^ and to finding novel compounds as treatments for the disease.^[^ ^17^ ^]^ As a result of this, in addition to the few treatments approved, a substantial number of new structures have been screened against targets of relevance for the disease, both virtually and in the wet lab.^[^ ^18^, ^19^, ^20^ ^]^ Even though these compounds might never reach the clinical phase for COVID‐19, they still hold value for other therapeutic uses, in special, for neglected diseases, as many parasitic targets share similarities to enzymes that are relevant for COVID‐19.

In this work, we highlight the potential of this chemical matter by leveraging ML models to identify, from a library of compounds originally designed as covalent inhibitors of targets relevant to SARS‐CoV‐2 infection (Patent Application BR 10 2024 016304‐4), compounds that present high inhibitory affinity against cruzain, the main cysteine protease of the parasite *Trypanosoma cruzi*, and a validated target for the treatment of Chagas disease.^[^ ^21^ ^]^ We also characterize the thermodynamic interaction of some of those hits with the target by using isothermal titration calorimetry (ITC) and discuss these results with computational and statistical analyses.

## 2 Experimental Section

### 2.1 ML Models

#### 2.1.1 Data Collection

The dataset used for training the models was sourced from publicly available chemical databases. The model presented here was first developed in 2021 and has been updated as more data becomes available. The version used in this work used data downloaded from BindingDB and ChEMBL (version 33) in 18/07/2023. Only compounds with potencies measured as *K*_i_ and IC_50_ and Standard units measured as “nM” were selected. Compounds from our internal library will be referred to using their internal codes (consisting in the word “Neq” followed by four numbers) to facilitate discussion within SAR analyses.

We opted to train classification models and used a “*p*Activity” (negative base 10 log of the activities in M units) cutoff value of 6.0 to classify compounds as active or inactive, considering our previous experience with cruzain inhibitors. As such, compounds with exact activity values or with censored activities greater than 1000 nM or smaller than 1000 nM were added to the dataset. *K*_i_ values were multiplied by 2.3 prior to conversion to *p*Activity values.^[^ ^22^ ^]^ Since compounds with *p*Activity < 3 or > 12 are generally regarded as outside the usual range of potencies for enzyme inhibitors, they were removed from the dataset. Finally, the binary activity labels were generated, with compounds with *p*Activity >= 6.0 being considered as “Active”, and the remaining, as “Inactive”.

Compounds were represented as SMILES strings, along with the corresponding bioactivity labels. Standardization of the structures was performed with the Python (version 3.10.6) module chembl_structure_pipeline (version 1.2.0), which includes steps such as normalization of chemotypes and removal of counterions and mixtures. Compounds containing “nonorganic” atoms were removed. Duplicated entries were identified by generating Morgan fingerprints (radius = 3, size = 1024) with the RDKit module (version 2023.03.2); entries that generated the same fingerprint were considered as duplicates, and only the one with the highest *p*Activity value was kept. Finally, compounds with a molecular weight < 200 and > 1000 were removed. The final cruzain inhibitors dataset contained 857 compounds, with 343 belonging to the “Active” class, represented as 1, and 514 to the “Inactive” class, represented as 0. RDKit's implementation of Morgan fingerprints (radius = 3, size = 1024) was chosen as the form of molecular representation for training the models. Data preprocessing was performed on Jupyter Notebooks, available as Supporting Information and in a GitHub repository (https://github.com/rflameiro/cmdc_202500630).

#### 2.1.2 Model Training

Classification models were trained using the automated ML (AutoML) Python module PyCaret (version 3.0.4). Within PyCaret, the dataset was split into a training and a test set (80:20) using a stratified sampling approach to maintain class distribution. The function compare_models() was used to evaluate and compare 15 different classification algorithms with fivefold cross‐validation and the following metrics were calculated: Accuracy, area under the receiver operating characteristic curve (AUC‐ROC), Recall, Precision, F1‐score, Cohen's Kappa, Matthew's correlation coefficient (MCC), and area under the precision–recall curve (AUPRC). Default hyperparameters were used. The metric MCC was selected to sort the models trained, and the model with the highest MCC was chosen for further validation.

Quality metrics and the corresponding confidence intervals were recalculated for the selected model using 10‐fold cross‐validation. The final model was trained using the full training set and further evaluated on the remaining 20% dataset (test set) to assess its generalizability. Additionally, 10 runs of y‐randomization were performed to confirm the absence of chance correlations; this involved shuffling the activity labels and retraining the model to verify that predictive performance significantly degraded, indicating that a true structure–activity relationship was captured by the model.

### 2.2 Enzyme Inhibition

The enzymatic activity was evaluated by fluorimetric assays on a Biotek Synergy HT equipment (Agilent, CA, USA) monitoring the hydrolysis rate of the fluorogenic substrate Z‐Phe‐Arg‐7‐amido‐4‐methylcoumarin (Z‐FR‐MCA, Sigma‐Aldrich, MA, USA) with fluorescence emission at 460 nm and excitation at 355 nm. Details are provided as Supporting Information.

### 2.3 Synthesis

#### 2.3.1 Chemistry

Chemical reagents were purchased from Sigma‐Aldrich (MA, USA), Combi‐Blocks (CA, USA), and ChemScene (NJ, USA), with at least 95% purity, and used as received without further purification, unless otherwise stated. All solvents were dried and distilled before use by standard procedures. ^1^H and ^13^C nuclear magnetic resonance (NMR) spectra were recorded on Agilent spectrometers (CA, USA), models 400/54 and 500/54, operating at 400 MHz for ^1^H (101 MHz for ^13^C) or 500 MHz for ^1^H (126 MHz for ^13^C). Chemical shifts (*δ*) are reported relative to the internal standard TMS (*δ* = 0.0 for ^1^H and ^13^C) or to the solvent residual signal (CDCl_3_: 7.26 ppm for ^1^H and 77.16 ppm for ^13^C; DMSO‐*d*6: 2.50 ppm for ^1^H and 39.52 ppm for ^13^C). High‐resolution mass spectra (HRMS) were obtained on a Thermo Scientific LTQ Velos Orbitrap spectrometer (MA, USA) operating in electrospray ionization conditions; values reported are the exact mass calculated using ChemDraw Professional 17.0 and mass found. Melting points were determined in a Microquímica Equipamentos (Palhoça, SC, Brazil) equipment, model MQAPF‐302, and are not corrected. Purity analysis and chromatographic purification were performed on a Shimadzu Prominence HPLC system (Shimadzu Corp., Kyoto, Japan) coupled to a Bruker (MA, USA) Amazon Ion Trap mass spectrometer operating in electrospray ionization conditions. The HPLC system was equipped with an LC‐20AT/AD ternary pumping system, a SIL‐20A autosampler, and a CTO‐20A column oven (Shimadzu Corp.). The column used for purity analysis was a ChiralpakR IC 5.0 μm (4.6 × 250.0 mm, Diacel Corporation, PA, USA). Gradient elution conditions were used, with a mobile phase composed of water (A) and acetonitrile (B), at a flow rate of 0.5 ml min^−1^. The gradient was set as follows: from 0.00 to 30.00 min, B was increased from 5% to 100%; from 30.01 to 40.0 min, B was kept at 100%; at 40.01 min, B was decreased to 5%, and from 40.01 to 50.00 min, B was held at 5%. Low‐resolution mass spectra were obtained at a capillary voltage of 4500 V, the capillary temperature was set at 300 °C, the pressure of the nebulizer was 35 psi, and the dry gas flow was 9 ml min^−1^.

Reactions in nonaqueous solvents were performed under argon atmosphere, and the glassware was dried at 120 °C. Dichloromethane and tetrahydrofuran were distilled with calcium hydride and benzophenone. Reactions were monitored using Sigma‐Aldrich thin‐layer chromatography plates (silica‐gel over aluminum foils, with fluorescent indicator) and spotted under UV light (254 nm), followed by staining with ethanolic 5% vanillin solution or ethanolic 5% phosphomolybdic acid solution.

Purification of compounds was performed by column chromatography on silica‐gel, with mixtures of ethyl acetate and hexane as mobile phase. When necessary, recrystallization from a dichloromethane/hexane solution was performed on the final products. For the products that did not present purity greater than 95%, purification using a semipreparative chiral column was performed.

**Scheme 1:**
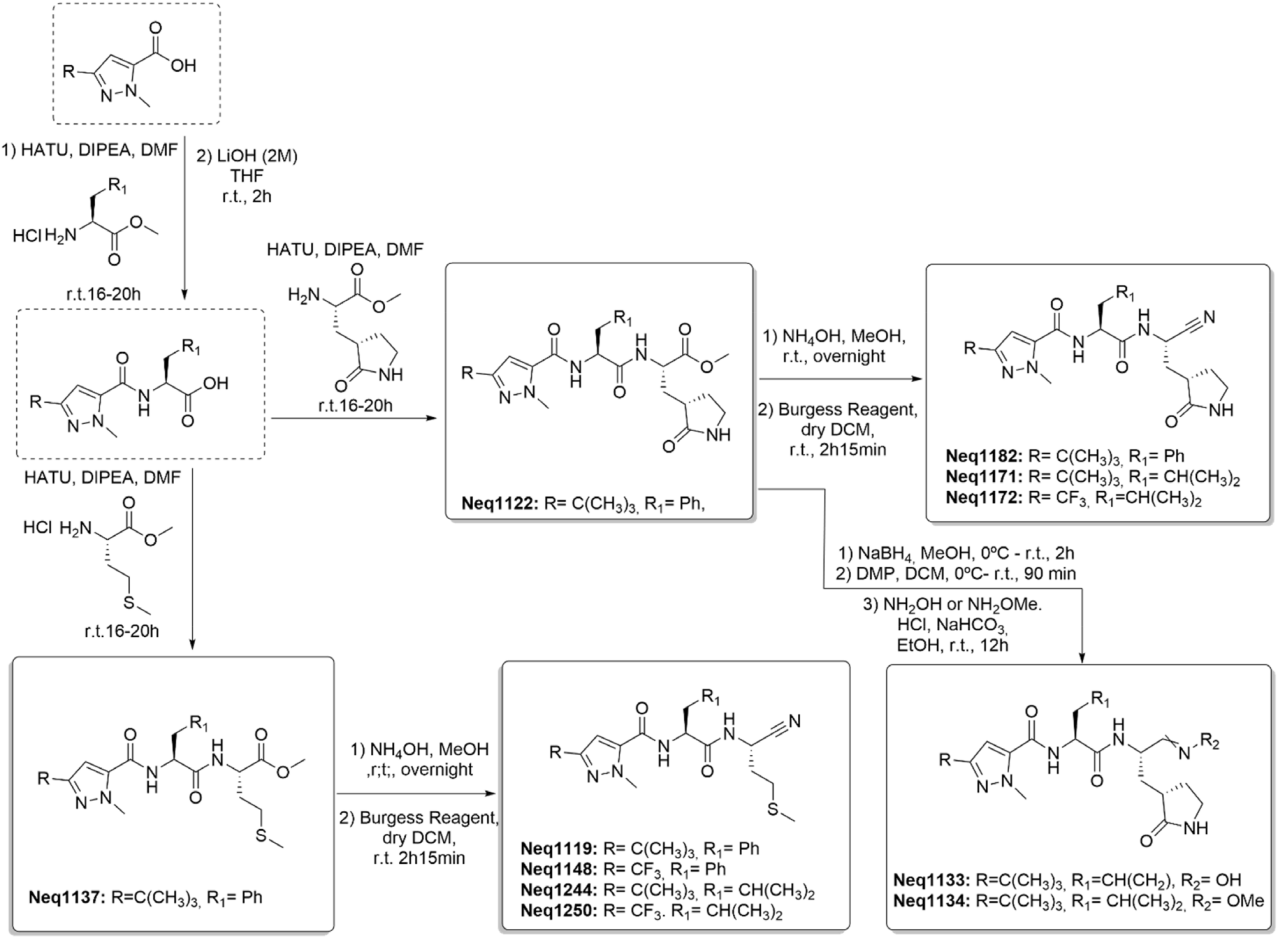
Routes followed for synthesis of peptidomimetic compounds.

**Scheme** **1** summarizes the synthetic routes used. Detailed procedures, compound codes and corresponding molecular structures are provided as Supporting Information.

#### 2.3.2 3‐(tert‐butyl)‐N‐((S)‐1‐(((S)‐1‐cyano‐3‐(methylthio)propyl)amino)‐1‐oxo‐3‐phenylpropan‐2‐yl)‐1‐methyl‐1H‐pyrazole‐5‐carboxamide (Neq1119)

White solid, melting point 67.9–68.9 °C. ^1^H NMR (400 MHz, CD_3_OD) *δ* 7.33–7.20 (m, 5H), 6.64 (s, 1H), 4.99–4.93 (m, 1H), 4.65 (dt, *J* = 15.6, 12.0 Hz, 2H), 3.93 (s, 3H), 3.34 (s, 1H), 3.19 (dd, *J* = 13.6, 7.1 Hz, 1H), 3.03 (dd, *J* = 13.6, 8.4 Hz, 1H), 2.57 (td, *J* = 7.2, 3.9 Hz, 2H), 2.12–2.02 (m, 5H), 1.28 (s, 9H). ^13^C NMR (101 MHz, CD_3_OD) *δ* 171.96, 160.57, 160.13, 136.66, 135.21, 128.92(2C), 128.16(2C), 126.52, 117.75, 103.59, 54.89, 38.94, 37.25, 37.07, 31.47, 31.37, 29.43(3C), 28.89, 13.67. MS: m/z calcd. for C_23_H_31_N_5_O_2_S+H^+^: 442.23; found: 442.27. Purity > 95%.

#### 2.3.3 Methyl (S)‐2‐((S)‐2‐(3‐(tert‐butyl)‐1‐methyl‐1H‐pyrazole‐5‐carboxamido)‐3‐phenylpropanamido)‐3‐((S)‐5‐oxopyrrolidin‐3‐yl)propanoate (Neq1122)

White solid, melting point: 106.3–107.1 °C. ^1^H NMR (500 MHz, CD_3_OD) *δ* 7.32–7.26 (m, 4H), 7.24–7.20 (m, 1H), 6.62 (s, 1H), 4.79 (dd, *J* = 9.3, 5.7 Hz, 1H), 4.54 (dd, *J* = 11.5, 4.0 Hz, 1H), 3.92 (s, 3H), 3.71 (s, 3H), 3.28–3.23 (m, 2H), 3.03 (dd, *J* = 13.9, 9.4 Hz, 1H), 2.53 (ddd, *J* = 18.8, 10.2, 4.2 Hz, 1H), 2.35–2.28 (m, 1H), 2.21–2.13 (m, 2H), 1.86–1.75 (m, 2H), 1.29 (s, 9H). ^13^C NMR (126 MHz, CD_3_OD) *δ* 180.31, 172.49, 172.05, 160.61, 160.13, 137.06, 135.40, 128.95, 128.03, 126.38, 103.532, 54.79, 51.46, 50.62, 38.13, 37.21, 37.06, 32.47, 29.45, 29.36, 29.33, 27.31. HRMS: m/z calcd. for C_26_H_35_N_5_O_5_+H^+^: 498.2711; found: 498.2727. Purity > 95%.

#### 2.3.4 3‐(tert‐butyl)‐N‐((S)‐1‐(((S)‐1‐(hydroxyimino)‐3‐((S)‐2‐oxopyrrolidin‐3‐yl)propan‐2‐yl)amino)‐4‐methyl‐1‐oxopentan‐2‐yl)‐1‐methyl‐1H‐pyrazole‐5‐carboxamide (Neq1133)

White solid, melting point: n.d. (mixture of diastereoisomers). ^1^H NMR (400 MHz, methanol‐*d*4): *δ* 7.32 (d, *J* = 5.3 Hz, 1H), 6.76 (d, *J* = 2.7 Hz, 1H), 6.62 (d, *J* = 5.8 Hz, 1H), 5.17 (ddd, *J* = 11.5, 5.9, 3.6 Hz, 1H), 4.65 (ddd, *J* = 11.2, 5.3, 4.2 Hz, 1H), 4.49 (ddd, *J* = 9.6, 5.2, 3.5 Hz, 1H), 4.02 (d, *J* = 1.3 Hz, 3H), 3.29–3.21 (m, 1H), 2.62–2.50 (m, 1H), 2.42–2.29 (m, 1H), 2.16 (s, 1H), 2.08–1.99 (m, 1H), 1.95–1.75 (m, 1H), 1.75–1.69 (m, 2H), 1.68–1.59 (m, 2H), 1.31 (s, 9H), 0.98 (ddd, *J* = 12.1, 6.2, 1.3 Hz, 6H). ^13^C NMR (101 MHz, methanol‐*d*4): *δ* 208.66, 180.86, 180.81, 173.45, 173.33, 160.85, 160.82, 160.18, 150.32, 149.04, 135.37, 103.61, 103.59, 52.23, 43.66, 40.18, 40.15, 40.11, 40.07, 38.17, 37.99, 37.36, 33.61, 32.25, 31.49, 24.70, 24.68, 21.95, 21.94, 20.56. MS: m/z calcd. for C_22_H_36_N_6_O_4_+Na^+^: 471.27; found: 471.29. Purity > 98%.

#### 2.3.5 3‐(tert‐butyl)‐N‐((S)‐1‐(((S)‐1‐(methoxyimino)‐3‐((S)‐2‐oxopyrrolidin‐3‐yl)propan‐2‐yl)amino)‐4‐methyl‐1‐oxopentan‐2‐yl)‐1‐methyl‐1H‐pyrazole‐5‐carboxamide (Neq1134)

White solid, melting point: n.d. (mixture of diastereoisomers). ^1^H NMR (400 MHz, CDCl_3_): *δ* 7.93 (d, *J* = 6.6 Hz, 1H), 7.34 (d, *J* = 4.6 Hz, 1H), 6.65 (m, *J* = 8.8 Hz, 1H), 6.45 (d, *J* = 0.6 Hz, 1H), 4.70 (m, *J* = 8.5, 5.2 Hz, 1H), 4.56 (m, *J* = 11.1, 6.6, 4.4 Hz, 1H), 4.08 (d, *J* = 0.6 Hz, 3H), 3.90–3.76 (m, 3H), 3.40–3.28 (m, 2H), 2.49–2.34 (m, 2H), 2.19–2.09 (m, 1H), 1.91–1.79 (m, 1H), 1.79–1.69 (m, 2H), 1.61 (dd, *J* = 8.8, 7.6 Hz, 1H), 1.33–1.27 (m, 9H), 0.98 (dd, *J* = 6.3, 2.7 Hz, 6H). ^13^C NMR (101 MHz, CDCl_3_): *δ* 180.03, 172.36, 160.25, 159.74, 149.12, 134.89, 102.95, 61.72, 51.46, 48.58, 42.72, 40.48, 38.92, 38.32, 33.44, 31.93, 30.49, 29.66, 28.91, 24.84, 22.84, 22.23. MS: m/z calcd. for C_23_H_38_N_6_O_4_+H^+^: 485.28; found: 485.31. Purity > 97%.

#### 2.3.6 Methyl (3‐(tert‐butyl)‐1‐methyl‐1H‐pyrazole‐5‐carbonyl)‐L‐phenylalanyl‐L‐methioninate (Neq1137)

White solid, melting point: 101.7–102.5 °C. ^1^H NMR (400 MHz, CDCl_3_) *δ* 7.35–7.20 (m, 5H), 6.68 (d, *J* = 7.6 Hz, 1H), 6.49 (d, *J* = 7.6 Hz, 1H), 6.29 (s, 1H), 4.78 (td, *J* = 7.6, 6.2 Hz, 1H), 4.61 (td, *J* = 7.4, 5.2 Hz, 1H), 4.05 (s, 3H), 3.72 (s, 3H), 3.15 (ddd, *J* = 21.4, 13.8, 6.9 Hz, 2H), 2.40 (t, *J* = 7.5 Hz, 2H), 2.08 (dt, *J* = 15.2, 7.6 Hz, 1H), 2.01 (s, 3H), 1.92 (dt, *J* = 21.7, 7.2 Hz, 1H), 1.26 (s, 9H). ^13^C NMR (101 MHz, CDCl_3_) *δ* 171.55, 170.40, 160.29, 159.83, 136.12, 134.57, 129.34, 128.75, 127.23, 103.05, 54.39, 52.58, 51.76, 38.88, 38.45, 31.91, 31.25, 30.45, 29.77, 15.37. HRMS: m/z calcd. for C_24_H_34_N_4_O_4_S+H^+^: 475.2374; found: 475.2395. Purity > 98%.

#### 2.3.7 N‐((S)‐1‐(((S)‐1‐cyano‐3‐(methylthio)propyl)amino)‐1‐oxo‐3‐phenylpropan‐2‐yl)‐1‐methyl‐3‐(trifluoromethyl)‐1H‐pyrazole‐5‐carboxamide (Neq1148)

Off‐white solid, melting point: 127–129 °C. ^1^H NMR (400 MHz, DMSO‐*d*6) *δ* 7.32–7.21 (m, 5H), 7.18–7.13 (m, 1H), 4.85 (d, *J* = 6.3 Hz, 1H), 4.60 (ddd, *J* = 10.1, 8.1, 4.9 Hz, 1H), 3.98 (s, 3H), 3.11 (dd, *J* = 13.7, 4.9 Hz, 1H), 2.92 (dd, *J* = 13.7, 10.2 Hz, 1H), 2.50 (ddd, *J* = 6.7, 4.0, 1.6 Hz, 2H), 2.02 (s, 6H). ^13^C NMR (101 MHz, DMSO‐*d*6) *δ* 171.24, 158.59, 139.20, 137.98, 137.12, 129.53, 128.63, 126.91, 122.87, 119.35, 106.51, 54.85, 39.47, 37.14, 31.61, 29.18, 14.90. HRMS: m/z calcd. for C_20_H_22_F_3_N_5_O_2_S+H^+^: 454.1519; found: 454.1507. Purity > 96%.

#### 2.3.8 3‐(tert‐butyl)‐N‐((S)‐1‐(((S)‐1‐cyano‐2‐((S)‐2‐oxopyrrolidin‐3‐yl)ethyl)amino)‐4‐methyl‐1‐oxopentan‐2‐yl)‐1‐methyl‐1H‐pyrazole‐5‐carboxamide (Neq1171)

White solid, melting point: 109.2–109.9 °C. ^1^H NMR (400 MHz, CDCl_3_) *δ* 8.66 (d, *J* = 6.3 Hz, 1H), 6.78 (d, *J* = 8.6 Hz, 1H), 6.55 (s, 1H), 6.48 (s, 1H), 4.76 (ddd, *J* = 14.0, 10.0, 5.8 Hz, 2H), 4.07 (s, 3H), 3.44–3.29 (m, 2H), 2.49–2.34 (m, 3H), 1.92 (ddd, *J* = 19.3, 8.9, 6.7 Hz, 2H), 1.77–1.62 (m, 3H), 1.29 (s, 9H), 0.98 (dd, *J* = 5.8, 2.9 Hz, 6H). ^13^C NMR (101 MHz, CDCl_3_) *δ* 179.05, 172.69, 160.25, 159.89, 134.76, 118.14, 103.28, 51.24, 42.03, 40.57, 39.46, 38.91, 38.07, 33.35, 31.95, 30.42, 28.23, 24.82, 22.85, 22.02. HRMS: m/z calcd. for C_22_H_34_N_6_O_3_ = 431.2765; found: 431.2798. Purity > 96%.

#### 2.3.9 N‐((S)‐1‐(((S)‐1‐cyano‐2‐((S)‐2‐oxopyrrolidin‐3‐yl)ethyl)amino)‐4‐methyl‐1‐oxopentan‐2‐yl)‐1‐methyl‐3‐(trifluoromethyl)‐1H‐pyrazole‐5‐carboxamide (Neq1172)

White solid, melting point: 99–101 °C. ^1^H NMR (400 MHz, CDCl_3_) *δ* 6.03 (s, 1H), 5.91 (s, 1H), 5.36–5.33 (m, 1H), 4.93 (dd, *J* = 11.0, 4.4 Hz, 1H), 4.83–4.77 (m, 1H), 4.71–4.66 (m, 1H), 4.20 (d, *J* = 2.2 Hz, 1H), 3.82 (s, 1H), 3.77 (s, 1H), 3.68 (s, 1H), 3.44 (ddd, *J* = 24.0, 12.1, 5.7 Hz, 1H), 3.16 (qd, *J* = 7.4, 4.8 Hz, 1H), 2.06 (s, 1H), 1.37 (t, *J* = 7.3 Hz, 1H), 1.26 (s, 2H), 1.02–0.95 (m, 1H), 0.85 (dd, *J* = 12.4, 8.7 Hz, 1H). ^13^C NMR (101 MHz, CDCl_3_) *δ* 177.70, 171.61, 159.73, 141.69, 133.34, 121.51, 116.07, 104.82, 52.24, 46.65, 41.92, 41.20, 40.39, 39.67, 29.86, 28.54, 23.12, 21.96. MS: m/z calcd. for C_19_H_25_F_3_N_6_O_3_+H^+^: 443.20; found: 443.25. Purity > 99%.

#### 2.3.10 3‐(tert‐butyl)‐N‐((S)‐1‐(((S)‐1‐cyano‐2‐((S)‐2‐oxopyrrolidin‐3‐yl)ethyl)amino)‐1‐oxo‐3‐phenylpropan‐2‐yl)‐1‐methyl‐1H‐pyrazole‐5‐carboxamide (Neq1182)

White solid, melting point: 113–114.5 °C. ^1^H NMR (500 MHz, CDCl_3_) *δ* 8.36 (d, *J* = 6.4 Hz, 1H), 7.36–7.18 (m, 5H), 6.83 (d, *J* = 8.2 Hz, 1H), 6.42 (s, 1H), 6.20 (s, 1H), 4.91 (dd, *J* = 14.7, 6.6 Hz, 1H), 4.75 (dt, *J* = 11.6, 5.9 Hz, 1H), 4.04 (s, 3H), 3.41–3.30 (m, 2H), 3.17 (qd, *J* = 13.7, 6.4 Hz, 2H), 2.42–2.17 (m, 3H), 2.01–1.79 (m, 2H), 1.28 (s, 9H). ^13^C NMR (126 MHz, CDCl_3_) *δ* 179.07, 171.13, 160.31, 159.78, 135.79, 134.60, 129.54, 128.68, 127.19, 117.79, 103.34, 53.97, 40.57, 39.52, 38.89, 38.82, 38.01, 33.30, 31.93, 30.47, 29.68, 28.31. HRMS: m/z calcd. for C_25_H_32_N_6_O_3_+H^+^ 465.2609; found: 465.2656. Purity > 96%.

#### 2.3.11 3‐(tert‐butyl)‐N‐((S)‐1‐(((S)‐1‐cyano‐3‐(methylthio)propyl)amino)‐4‐methyl‐1‐oxopentan‐2‐yl)‐1‐methyl‐1H‐pyrazole‐5‐carboxamide (Neq1244)

Light yellow solid, melting point: 54.6–55.7 °C. ^1^H NMR (500 MHz, CDCl_3_) *δ* 7.12 (d, *J* = 8.2 Hz, 1H), 6.38 (s, 1H), 6.35 (d, *J* = 7.9 Hz, 1H), 5.09–5.03 (m, 1H), 4.56–4.49 (m, 1H), 4.09 (s, 3H), 2.65 (td, *J* = 6.9, 2.0 Hz, 2H), 2.16–2.09 (m, 2H), 2.08 (s, 2H), 1.80–1.67 (m, 3H), 1.29 (s, 9H), 0.98 (dd, *J* = 12.1, 6.3 Hz, 6H). ^13^C NMR (126 MHz, CDCl_3_) *δ* 171.32, 160.50, 160.48, 134.09, 117.75, 103.08, 51.46, 40.39, 39.81, 39.06, 31.70, 30.47, 29.67, 24.81, 22.81, 22.08, 15.43. HRMS: m/z calcd. for C_20_H_33_N_5_O_2_S+H^+^: 408.2428; found: 408.2430. Purity > 98%.

#### 2.3.12 N‐((S)‐1‐(((S)‐1‐cyano‐3‐(methylthio)propyl)amino)‐4‐methyl‐1‐oxopentan‐2‐yl)‐1‐methyl‐3‐(trifluoromethyl)‐1H‐pyrazole‐5‐carboxamide (Neq1250)

Yellow oil, melting point: n.d. ^1^H NMR (500 MHz, CDCl_3_) *δ* 7.45 (d, *J* = 8.0 Hz, 1H), 7.22 (d, *J* = 8.1 Hz, 1H), 6.91 (s, 1H), 5.05 (dt, *J* = 8.0, 7.0 Hz, 1H), 4.59–4.53 (m, 1H), 4.16 (s, 3H), 2.67 (m, *J* = 6.9, 4.3 Hz, 2H), 2.15 (m, *J* = 7.0, 2H), 2.11 (s, 3H), 1.72 (m, *J* = 6.1, 2.2 Hz, 3H), 0.98–0.95 (dd, 6H). ^13^C NMR (126 MHz, CDCl_3_) *δ* 171.87, 159.08, 140.88, 135.59, 121.75, 117.73, 105.4, 51.82, 40.64, 40.15, 39.97, 31.48, 29.68, 29.64, 24.83, 22.78, 21.92, 15.43, 15.36. HRMS: m/z calcd. for C_17_H_24_F_3_N_5_O_2_S+H^+^: 420.1676, found: 420.1674. Purity > 95%.

### 2.4 Thermodynamic Characterization

The ITC method was used to determine the thermodynamic profiles of selected cruzain inhibitors. The calorimetric assays were performed at 25 °C on a VP‐ITC microcalorimeter (Malvern Panalytical, Malvern, UK). Protein and inhibitor solutions were prepared with the same buffer used in the dialysis, adding 5% v/v DMSO and 0.001% Triton‐X 100 (Sigma‐Aldrich, MA, USA) to prevent protein aggregation. Inhibitor concentration was fixed at 0.16 nM and protein concentration varied from 20 to 35 µM, depending on the inhibitor's affinity: for high‐affinity inhibitors, the protein concentration was increased, otherwise, the concentration was decreased to ensure that saturation of the protein's active sites occurred gradually. The inhibitor was placed in the syringe and the protein in the cell. All thermodynamic parameters were calculated with Microcal PEAQ‐ITC Analysis Software and experiments were performed in duplicates. All the thermodynamic fingerprints discussed in this work are presented as Supporting Information.

## 3 Computational Methods

### 3.1 Molecular Docking

#### 3.1.1 System

All docking runs were made on Linux (Ubuntu 20.04 LTS), in a Python 3.12.9 environment.

#### 3.1.2 Receptor and Ligand Preparation

The structure for cruzain was obtained from the Protein Data Bank (PDB ID: 2OZ2) and processed with ChimeraX (on Windows) to remove water molecules and the bound ligand. Only chain C was retained for docking studies. The resulting structure was moved to the Linux environment and processed using the script mk_prepare_receptor.py (Meeko, version 0.6.1) to generate the necessary input files for docking.

3D sdf files for the ligands were prepared with RDKit and AutoDockTools. The files were processed using the scripts scrub.py and mk_prepare_ligand.py, to generate the pdbqt files for docking with AutoDock Vina.

#### 3.1.3 Docking Protocol

Docking was performed following the guidelines provided in the AutoDock Vina documentation (https://autodock‐vina.readthedocs.io/en/latest/docking_basic.html). AutoDock Vina (version 4.2.6) was used for docking, with the following parameters: Scoring function: AD4; Exhaustiveness: 32; Grid box size: 20 × 20 × 20 Å.

#### 3.1.4 Hydrated Docking

For hydrated docking, the protocol described in the AutoDock Vina documentation (https://autodock‐vina.readthedocs.io/en/latest/docking_hydrated.html) was followed. The scripts used can be found in https://github.com/ccsb‐scripps/AutoDock‐Vina/blob/develop/example/autodock_scripts/. The water map for cruzain was generated using the script mapwater.py and AutoGrid (version 4.2.9). Ligands were prepared with mk_prepare_ligand.py using the flag ‐w.

#### 3.1.5 Analysis

Results of the docking runs were saved in log files and interpreted. The script dry.py was used to interpret the results of the hydrated docking. Additionally, the resulting highest scoring poses were visually analyzed using ChimeraX.

## 4 Results and Discussion

### 4.1 ML‐Guided Selection of Potential Cruzain Inhibitors

After processing cruzain activity data, a binary dataset consisting of 343 “Active” and 514 “Inactive” compounds was obtained.

Results from the AutoML framework PyCaret showed good performances for models of different classes, which reflects the representation power of Morgan fingerprints for this molecular property prediction task. Morgan fingerprints are fixed‐size binary vectors resulting from the application of the Morgan algorithm to molecular structures in which positive bits indicate the presence of a certain molecular substructure in the molecule. As such, they tend to perform well in tasks involving molecular similarity like predictive QSAR models.^[^ ^23^ ^]^ One limitation of Morgan fingerprints is that they cannot distinguish between enantiomers or diastereomers; this is why one of the preprocessing steps in this work involved removing duplicated entries after calculating the fingerprints.

As is recommended in the literature,^[^ ^24^ ^]^ several metrics were calculated during model evaluation. However, considering the slight imbalance of the dataset, and that the model should balance the importance of both “Active” and “Inactive” classes, the metric MCC was selected to sort the models produced by PyCaret.^[^ ^25^ ^]^ Therefore, the model resulting from the use of the ExtraTrees (Extremely randomized trees, ET) algorithm was selected, as it presented the highest value of MCC, along with robust values for the other metrics. We are not claiming that the ranking by MCC is guaranteed to choose the best model architecture, as simply comparing cross‐validation results without considering the variance of the metrics and the limited dataset used does not constitute a statistically robust way of selecting a top performing model.^[^ ^26^ ^]^ As a matter of fact, ET was closely followed by random forest and gradient boosting classifier, other tree‐based algorithms that certainly also produced excellent models. Our only claim is that the chosen model, regardless of the specific model architecture, showed acceptable cross‐validation performance on all metrics and is a solid choice for screening a library of compounds.

The ET algorithm was then selected, and the model was reassessed via 10‐fold cross‐validation, with metrics calculated along with the corresponding confidence intervals (1.96 times the standard deviation for the 10 runs). We opted to use 10‐fold CV instead of the 5‐fold employed by PyCaret to allow the models to be trained on more data and better estimate the performance of the model with the entire dataset. Then, a final model was fit on the training data and validated on the external test set (20% of the original dataset). Finally, 10 rounds of y‐randomization were run to confirm that the model performance was not by chance. The results are reported in **Table** **1**.

**Table 1:** Metrics for 10‐fold cross‐validation, external evaluation on the test set and on the 10 rounds of y‐randomization for the ExtraTrees model.

Metric	10‐fold CV	External test set	y‐randomization
Accuracy	0.848 ± 0.062	0.837	0.547 ± 0.061
AUC‐ROC	0.905 ± 0.071	0.895	0.501 ± 0.067
Recall	0.763 ± 0.090	0.710	0.317 ± 0.096
Precision	0.845 ± 0.106	0.860	0.415 ± 0.103
F1‐score	0.801 ± 0.079	0.778	0.359 ± 0.095
Kappa	0.679 ± 0.130	0.651	0.018 ± 0.133
MCC	0.682 ± 0.132	0.659	0.018 ± 0.137
AUPRC	0.871 ± 0.079	0.833	0.419 ± 0.052

As both internal and external validations indicated a robust performance of the model, a final ET model was then fit on the entire dataset and used to predict activity classes for a library containing 52 peptidomimetic compounds originally designed as potential dual inhibitors of SARS‐CoV‐2 main protease (M^pro^) and human cathepsin L (hCatL).

Of the 52 compounds, 23 were predicted to be active. A higher proportion of compounds predicted to be active could have been expected, considering that cruzain is a hCatL‐like enzyme. However, the fact that the compounds were also designed to target M^pro^ probably led to a difference in chemical space compared to known cruzain inhibitors.

Instead of performing the enzyme inhibition test on all compounds predicted as active, we instead used the model to calculate the predicted probability, a value between 0 and 1, with values closer to 1 indicating a higher likelihood of the compound belonging to the “Active” class. It has been shown that selecting compounds with higher predicted probabilities translates to a higher confidence, while also being a better indicator of whether the compound is within the model's applicability domain than novelty detection approaches, such as nearest neighbors.^[^ ^27^ ^]^ We therefore chose the cutoff value of 0.7, that is, compounds with a predicted probability greater than 70% of inhibiting cruzain were selected for further evaluation.

Of the 11 compounds selected by this method, three (Neq1139, Neq1140, and Neq1141) presented the highest scores. However, all three compounds are analogs of known cruzain inhibitors, such as Neq0659/Neq0820^[^ ^28^ ^]^ and Cz007/Cz008.^[^ ^29^ ^]^ Therefore, due to the lack of novelty, they were not selected for the enzyme inhibition assays. The eight remaining compounds were then evaluated along with two newly synthesized compounds, Neq1244 and Neq1250, which were synthesized to improve the structure–activity relationship analysis.

### 4.2 Cruzain Inhibition Assays

The results of the cruzain inhibition assays for the eight selected compounds are presented in **Table** **2**, along with three other compounds that were included to complete the following SAR analysis.

**Table 2:** Codes for selected compounds, their predicted probability of being active according to the model and the measured *pK*_i_ against cruzain.

Code	Predicted probability (Active)	*pK*_i_ cruzain
Neq1119	0.83	7.6
Neq1122	0.70	5.7
Neq1133	0.72	6.8
Neq1134	0.73	5.7
Neq1137	0.81	5.9
Neq1148a)	0.39	6.9
Neq1171	0.87	7.9
Neq1172	0.79	7.1
Neq1182	0.81	7.2
Neq1244b)	–	8.0
Neq1250b)	–	7.3

^a)^ This compound was not predicted as active by the model, but its potency was determined to complete the SAR analysis.

^b)^ These compounds were not in the library but were synthesized to complete the SAR analysis.

Assay results demonstrate a successful identification of potent cruzain inhibitors. Those results are in line with our previous works on ML for modeling cruzain activity, which have been demonstrating high quality metrics for predicting cruzain inhibitors.^[^ ^30^ ^,^ ^31^ ^]^ Three of the eight compounds selected presented an inhibition constant higher than 1.0 µM (*pK*_i_ < 6.0), but two of them, Neq1122 and Neq1137 are worth discussing: the compounds are precursors to covalent inhibitors and have an ester group in place of the electrophilic warhead, which is known to lead to weaker inhibitors. Nevertheless, they were considered for the inhibition assay just to confirm the expected result. The fact that the model was not able to identify them as noninhibitors can be explained by a lack of ester‐containing compounds on the training set. Therefore, the compounds were likely selected as potentially active based on other structural features of peptidomimetic compounds. This highlights the importance of having as much information about inactive compounds as possible on the training set, so that the model can avoid selecting false positives.

**Scheme 2:**
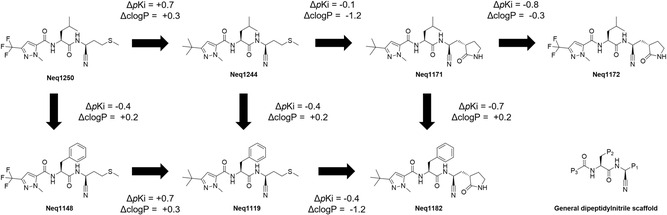
SAR analysis for the matched molecular series of seven compounds. At the bottom right corner, a general scaffold for a dipeptidylnitrile is shown.

Furthermore, we observed that four of the active compounds belonged to a matched molecular series and three additional compounds were included to complete the series: Neq1148, which was already in the library, and Neq1244 and Neq1250, which had not been previously synthesized. The complete series is presented in **Scheme** **2**, in which arrows represent one matched molecular transformation, and the corresponding changes in *pK*_i_ and clogP, calculated with RDKit. In the bottom right corner of Scheme 2, a dipeptidylnitrile scaffold indicates the positions of P3, P2, and P1 groups.

As is usual in SAR analyses, the change in inhibition activity (Δ*pK*_i_) will be discussed for all molecular pairs, which will be represented with the square bracket notation.^[^ ^32^ ^]^ For instance, the “transformation” of the trifluoromethyl group of Neq1250 to the tert‐butyl group in Neq1244 is noted as [Neq1250 → Neq1244].

**P3**: Replacing a tert‐butyl methyl pyrazole (TBMP) by a trifluoromethyl pyrazole (TFMP) on the P3 position changes *pK*_i_ by –0.7 log units in [Neq1250 → Neq1244], –0.7 log units in [Neq1148 → Neq1119], and –0.8 log units in [Neq1171 → Neq1172], while clogP decreases (–0.3 log units) for the three pairs. In general, the P3 position has a smaller impact on activity, considering that the cruzain S3 subsite is a mostly solvent‐exposed region.^[^ ^33^ ^,^ ^34^ ^]^ TBMP is considered a privileged building block for the inhibition of cruzain and hCatL^[^ ^35^ ^,^ ^36^ ^]^ and TFMP has been proposed as a derivative with improved pharmacokinetic properties, but the slight drop in *pK*_i_ suggests that this might not be a suitable strategy if optimizing for potency is of essence.

**P2**: Leucine derivatives (P2 = isobutyl) are slightly more potent than the phenylalanine counterparts (P2 = benzyl), and the Leu → Phe substitution changes *p*K_i_ by –0.4, –0.4, and –0.7 log units for [Neq1250 → Neq1148], [Neq1244 → Neq1119], and [Neq1171 → Neq1182], respectively. This is an expected result, as cruzain has been shown to have a slight preference for leucine‐based derivatives on the P2 position.^[^ ^37^ ^]^

**P1**: Replacing a methionine‐derivative by a γ‐lactam moiety in P1 has a very small impact on potency, with a Δ*pK*_i _= –0.1 for [Neq1244 → Neq1171] and –0.4 for [Neq1119 → Neq1182], and all three compounds with P1 = γ‐lactam have a *pK*_i_ > 7.0. The γ‐lactam (or pyrrolidinone) moiety is a polar R‐group that has been shown to lead to potent SARS‐CoV‐2 main protease inhibitors, such as nirmatrelvir^[^ ^15^ ^]^ and GC‐376,^[^ ^38^ ^]^ as it mimics the glutamine residue present in the enzyme's natural substrate, interacting with His163 (main chain) and Glu166 (main and side chains) in the S1 pocket. This R‐group has also been shown to interact with M^pro^ S2 residues in some aldehyde‐based inhibitors.^[^ ^39^ ^]^ This is a result of particular interest, considering that while dual inhibitors of SARS‐CoV‐2 M^pro^ and hCatL with the γ‐lactam in P1 have been reported in the literature,^[^ ^40^, ^41^, ^42^, ^43^ ^]^ to the best of our knowledge, this P1 moiety has never been reported in cruzain inhibitors. Moreover, these compounds, while highly potent against cruzain, have a significantly lower clogP than their analogs, which might lead to other improved properties, such as higher solubility and higher hERG IC_50_,^[^ ^44^ ^]^ to name a few. Given the potential of this new chemical matter, we tried to characterize the interaction between the γ‐lactam‐based ligands (and of some molecular pairs) and cruzain. For this, ITC was used to measure the free energy, enthalpy, and entropy of binding.^[^ ^45^ ^]^ The technique has proven useful in the characterization of protein‐ligand interactions for covalent inhibitors^[^ ^46^ ^]^ and for several targets,^[^ ^47^ ^,^ ^48^ ^]^ including cruzain.^[^ ^28^ ^,^ ^37^ ^,^ ^49^ ^,^ ^50^ ^]^

### 4.3 Thermodynamic Characterization of Novel Hits

**Scheme 3:**
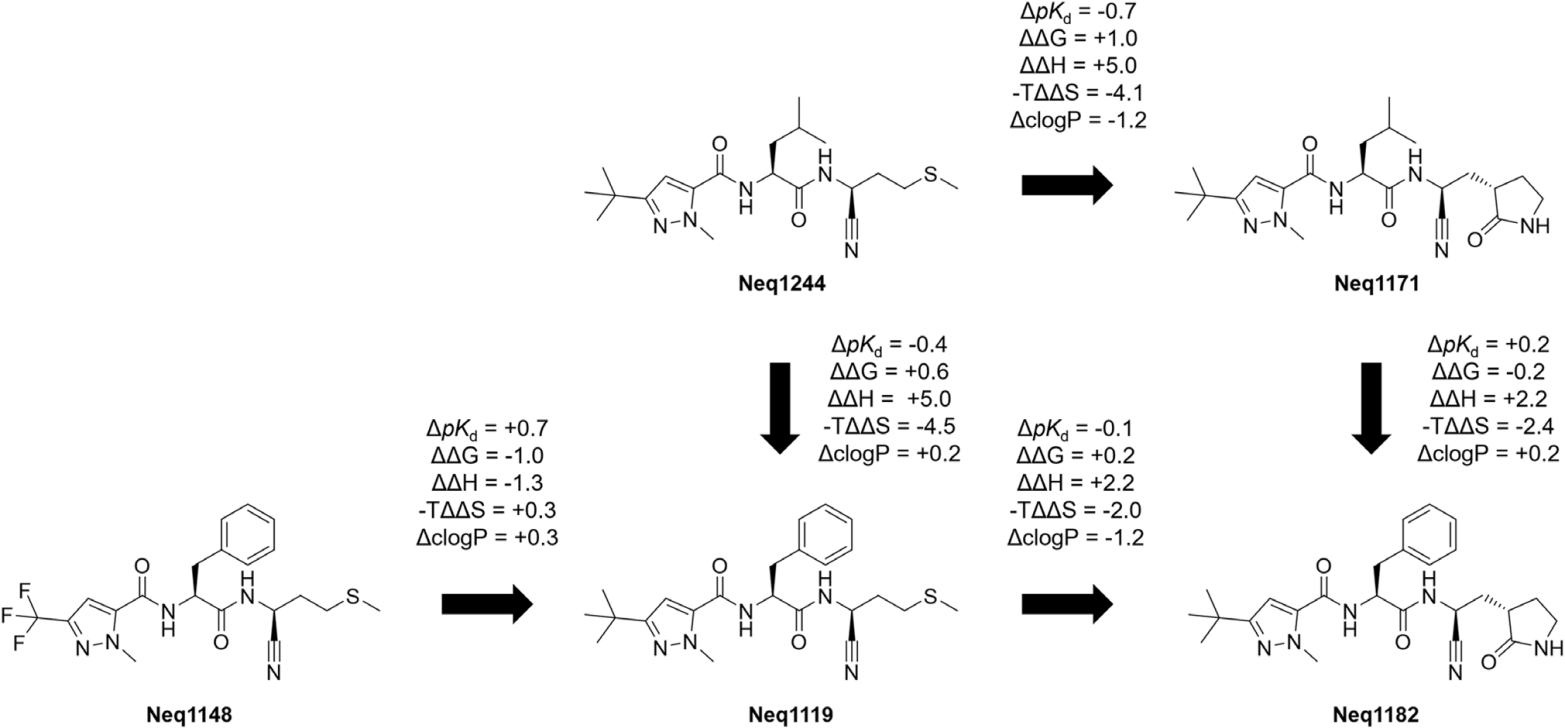
Matched molecular series with ITC results.

Five compounds were selected to build a matched molecular series for thermodynamic profiling with ITC. The thermodynamic fingerprints are presented in **Table** **3** and the matched molecular pairs (MMP) analysis is summarized in **Scheme** **3**. Due to rounding, the values of ΔG might not correspond exactly to the sum of ΔH and –TΔS.

**Table 3:** Thermodynamic fingerprints for five selected compounds.

Code	*pK*_d_	ΔG	ΔH	–TΔS
Neq1119	7.2	−9.8	−12.2	+2.4
Neq1148	6.5	−8.8	−10.9	+2.1
Neq1171	6.9	−9.4	−12.2	+2.8
Neq1182	7.1	−9.6	−10.0	+0.4
Neq1244	7.6	−10.4	−17.2	+6.9

#### 4.3.1 Enthalpy–Entropy Compensation (EEC)

**Figure 1:**
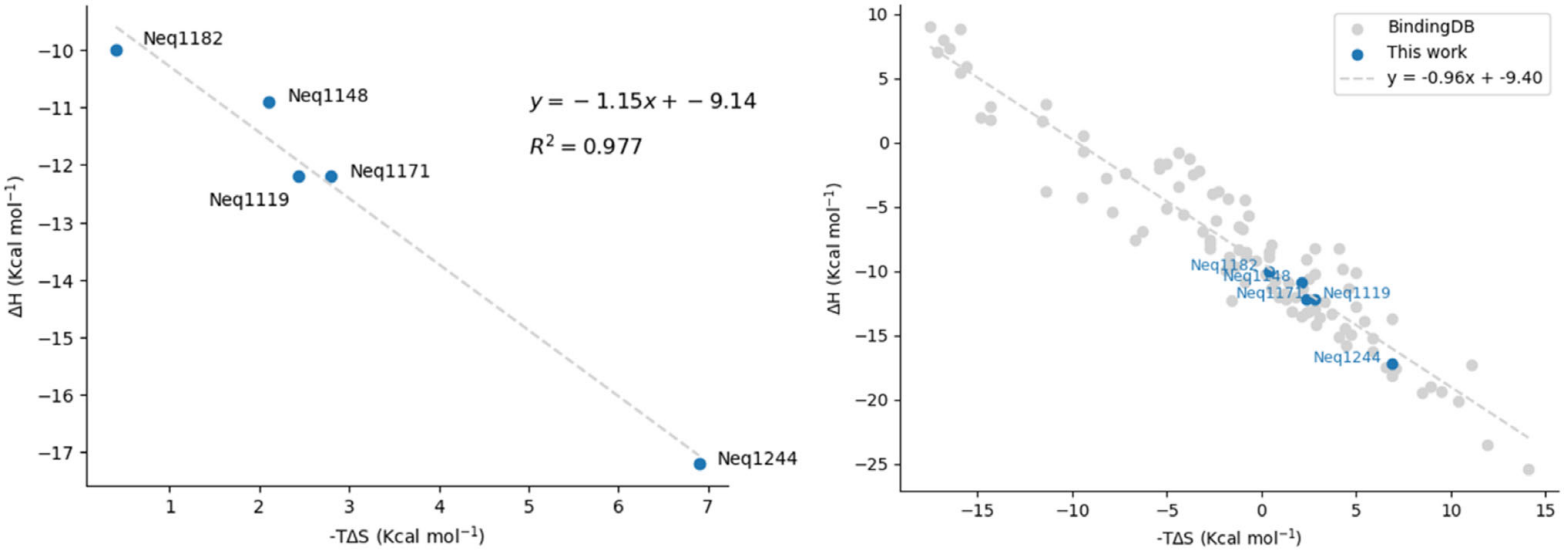
Plots of ΔH versus –TΔS demonstrating EEC for the five compounds in the molecular series (left) and with the inclusion of compounds selected from BindingDB (right).

A relevant result of the ITC characterization is that the most active compounds in our series do not show similar thermodynamic profiles despite having similar inhibition/dissociation constants. In other words, the potent compounds with the smallest entropic contributions also show a proportional drop in the enthalpic component. This phenomenon is well known in medicinal chemistry literature and is called the EEC effect, described as “a linear correlation between enthalpy and entropy changes in chemical processes where closely related structures or conditions are involved.”^[^ ^51^ ^]^ We can demonstrate this for our series by creating a plot of ΔH versus –TΔS, as shown in **Figure** **1** (left), which shows a linear correlation (R^2^ = 0.977).

Additionally, the results of this study were compared with a reference dataset extracted from the public BindingDB database (https://www.bindingdb.org). For this, the thermodynamic parameters of enthalpy (ΔH) and entropy (ΔS) of binding for 102 compounds, spanning 14 distinct molecular target classes, were used to construct the plot shown in Figure 1 (right). The experimental data points from the literature, in gray, demonstrate a linear correlation between –TΔS and ΔH, that is, the EEC effect. The blue data points, representing the results from the present study, align as expected with the trend of the series.

EEC is said to be a consequence of weak intermolecular interactions, as is the case of van der Waals interactions between the ligand and the protein, and hydrogen bonds in water. It is often explained in terms of a balance of factors, as stronger enthalpies of binding lead to a tighter system, which has less degrees of freedom and is less favorable entropically.^[^ ^52^ ^]^ In addition to the flexibility of the binding partners, the other main factor that affects EEC are solvent‐related effects, such as desolvation effects and the costly reorganization of water molecules at the binding interface.^[^ ^51^ ^]^

In essence, the main consequence of EEC is that enthalpy change (ΔH), in isolation, proves inadequate as a reliable predictor of binding affinity (ΔG). That is, even in cases where ΔH is strongly negative, suggesting the formation of highly favorable interactions, these enthalpic gains may be substantially, or even completely, negated by an opposing entropic penalty. This highlights a critical conceptual pitfall in drug design: relying on enthalpic optimization alone, without accounting for entropy, risks generating misleading conclusions and promoting inefficient design cycles. A ligand may appear promising based on enthalpy‐focused modeling yet ultimately fail to demonstrate a proportionally high inhibitory affinity due to entropic liabilities that were overlooked (e.g., the thermodynamic profile for Neq1244).

Considering both components of the free energy equation in the design of compounds presents a significant challenge, particularly given the elusive nature of entropy in molecular systems. Unlike enthalpy, which can often be decomposed into well‐characterized interaction terms, entropy arises from complex and dynamic phenomena that are difficult to quantify or modulate with precision using conventional structure‐based drug design approaches. Approaches that incorporate molecular dynamics, water thermodynamics, or ML‐based estimations of entropic contributions may offer a way forward.

#### 4.3.2 MMP Analysis of P3 Substitution

For [Neq1148 → Neq1119], replacing the trifluoro methyl pyrazole group (TFMP) by the tert‐butyl analog (TBMP) on the P3 position increases *pK*_d_ by + 0.7 log units, just as was observed for *pK*_i_. The free energy of binding changes by –1.0 kcal mol^−1^, but the entropic components are similar, therefore, the change in the enthalpy of binding is the main driver of the superior potency of Neq1119. Considering that increasing lipophilicity often favors binding, it is noteworthy that Neq1148, despite having the most lipophilic substituents in P1 and P2 in the series, not only does not show a favorable entropy component of binding but also has the lowest *pK*_d_ of the series.

#### 4.3.3 MMP Analysis of P2 Substitution

For [Neq1119 → Neq1244], replacing the P2 moiety from phenylalanine to leucine improves *pK*_d_ by +0.4 log units. The free energy of binding changes by –0.6 kcal mol^−1^, mainly driven by an improvement in the enthalpy of binding (–5.0 kcal mol^−1^). This, however, is compensated by a higher value of –TΔΔS (+4.5 kcal mol^−1^). The pair [Neq1182 → Neq1171], which involves the same substitution, has a near‐zero ΔΔG like the previous pair, but lower individual contributions: ΔΔH = –2.2 kcal mol^−1^ and –TΔΔS = +2.4 kcal mol^−1^.

At first, this might come as an unexpected result, considering that the benzyl group of phenylalanine is a more lipophilic substituent than isobutyl. However, as previously discussed, cruzain is known to have a preference for leucine‐derived inhibitors in the P2 position. The improved binding that explains the difference in enthalpy should also lead to a decrease in the conformational freedom of both ligand and enzyme, thereby leading to an unfavorable entropic compensation.

#### 4.3.4 MMP Analysis of P1 Substitution

The transformation from methionine to γ‐lactam in P1 could be analyzed for two pairs. Those transformations yielded some interesting results, considering that a favorable or near‐zero entropic contribution was observed for one of the γ‐lactam derivatives, Neq1182. This, in our experience, is a rare observation among peptidomimetic cruzain inhibitors, and could be a starting point for optimized inhibitors that “beat the EEC”, that is, highly potent inhibitors with high enthalpic contributions and no entropic loss.

As with *pK*_i_, replacing methionine by γ‐lactam decreased *pK*_d_ slightly for [Neq1119 → Neq1182], by –0.1 log units, but a more significant decrease of –0.7 log units was observed for [Neq1244 → Neq1171], which does not reflect the near‐zero change in *pK*_i_ for this pair.

For the individual thermodynamic contributions, the values are strikingly different. ΔΔG = +0.2 kcal mol^−1^ for [Neq1119 → Neq1182], with a +2.2 kcal mol^−1^ increase in ΔH being counterbalanced by a decrease of –2.0 kcal mol^−1^ in –TΔS. For [Neq1244 → Neq1171], on the other hand, ΔΔG = +1.0 kcal mol^−1^, a result of a higher ΔΔH = +5.0 kcal mol^−1^, compensated by a –TΔΔS =–4.1 kcal mol^−1^.

The results show that the replacement of methionine by γ‐lactam in P1 contributes unfavorably to the enthalpy of binding, but favorably to the entropic component. This was contrary to the expectations, considering that increasing the lipophilicity of enzyme inhibitors is usually associated with more favorable entropic contributions to binding in ITC measurements.^[^ ^53^ ^,^ ^54^ ^]^

Following the observation of this H/S anomaly on the P1 analyses, we postulated that molecular flexibility and water‐related effects could be involved. Therefore, even though explaining entropic effects is far from trivial, we decided to further investigate this phenomenon by docking the two matched pairs on cruzain, Neq1244/Neq1171 and Neq1119/Neq1182, with and without explicit water molecules.

### 4.4 Docking

#### 4.4.1 Docking Without Solvent

One of the first possible explanations for the observed H/S anomaly relates to the higher flexibility of the methionine side chain. That is, when it interacts with the S1 region amino acid residues, the chain can no longer rotate freely, which should make the entropic contribution less favorable. However, docking on cruzain showed that the torsional free energy for methionine‐containing ligands is only 0.3 kcal mol^−1^ higher for both pairs, considering the best‐scoring poses. In fact, the methionine side chain has only one extra rotatable bond compared to the γ‐lactam analog, and the loss in degrees of freedom upon binding should not be enough to explain the significant differences in the entropic component.

While flexibility is straightforward to model for ligands, modeling the flexibility of enzymes requires more computationally expensive simulations. Works considering the flexibility of cruzain to identify novel inhibitors have been proposed,^[^ ^55^ ^]^ but these seem to be more relevant when nonpeptidic compounds are being screened, as cruzain bound to peptidic compounds is known to be less flexible,^[^ ^56^ ^]^ mainly due to the formation of three hydrogen bonds to Gly66 and Asp161.^[^ ^36^ ^,^ ^57^ ^]^ Therefore, this hypothesis for the H/S anomaly was not assessed in this work.

#### 4.4.2 Hydrated Docking

Besides ligand‐ and protein‐related factors, another significant source of changes to the thermodynamic profiles of enzyme inhibitors is the distribution of solvent molecules. While the rearrangement of water molecules is harder to model and predict than flexibility,^[^ ^58^ ^]^ the displacement of high‐energy water molecules is a common mechanism used to improve the binding of enzyme inhibitors for which computational models have been developed. High‐energy water molecules are those with unfavorable interactions with an enzyme and, when displaced by a well‐placed substituent on the ligand, can lead to improvements in both enthalpy and entropy of binding.^[^ ^59^ ^,^ ^60^ ^]^

The docking software AutoDock Vina provides the option to perform docking runs with explicit water molecules. The “hydrated docking” works by adding “dummy” atoms with water‐like properties (H‐bond donor and H‐bond acceptor properties) around the ligands, then performing the docking of this ensemble on the enzyme. The algorithm then classifies the water molecules according to their impact on binding: when a ligand–enzyme interaction is energetically favorable and requires that a water be displaced, the water is marked as DISPLC, and the desolvation entropy resulting from its release contributes to the binding free energy. Conversely, when a favorable interaction is formed with the presence of the water, it is marked as STRONG or WEAK depending on the absolute value of the water–receptor enthalpy, which is then added to the binding free energy.^[^ ^61^ ^]^

Considering the best‐scoring docking poses, for the [Neq1244 → Neq1171] pair, replacing methionine by γ‐lactam does not change the number of DISPLC water, and there is one additional water marked as STRONG in Neq1171. For [Neq1119 → Neq1182], the γ‐lactam derivative Neq1182 contained two additional DISPLC and one less STRONG. Waters marked as DISPLC are expected to give a positive contribution to entropy (more negative –TΔS), while those marked as STRONG should contribute to H < 0 and S < 0. Therefore, while the results might point to a more favorable entropy of binding for Neq1182 due to water displacement, they are overall inconclusive, and do not suggest a robust explanation for the H/S anomaly for both pairs. This is not an entirely unexpected outcome, given that one study comparing specialized software for water prediction in binding sites showed that and their ability to reproduce water location and free energy was moderate.^[^ ^62^ ^]^ Hydrated docking is also an oversimplified analysis that models only the first shell of hydration.^[^ ^61^ ^]^ It is known that water forms networks that, when disturbed, can impact both entropic and enthalpic components, and processes far from the binding region can also impact binding. For instance, it has been observed that the release of water molecules can make more flexible ligands have a more favorable binding entropy.^[^ ^63^ ^]^

### 4.5 Comparing ITC Results to Other Dipeptidylnitriles

After failing to explain the anomalous behavior shown by some of our compounds with computational methods, we tried to determine whether those profiles were exclusive to those compounds or were present in other analogs as well. We observed that four of the compounds that had the thermodynamic fingerprint determined in this work have a scaffold corresponding to dipeptidylnitriles with a TBMP group at the P3 position. Other compounds with this same scaffold have been made in our laboratory^[^ ^32^ ^,^ ^36^ ^,^ ^64^ ^]^ and seven of them have also been analyzed with ITC.^[^ ^28^ ^]^ We included the thermodynamic fingerprints of these compounds, along with those reported in this paper, to conduct a comprehensive analysis and determine whether the methionine and γ‐lactam analogs follow the general trend of the series or represent outliers.

To facilitate discussion, the series were separated into two: compounds with a phenylalanine moiety in P2 (TBMP‐Phe‐P1‐CN series) and compounds with leucine in the same position (TBMP‐Leu‐P1‐CN series). The structures and ITC fingerprints of the additional compounds are presented as Supporting Information.

#### 4.5.1 TBMP‐Phe‐P1‐CN Series

**Figure 2:**
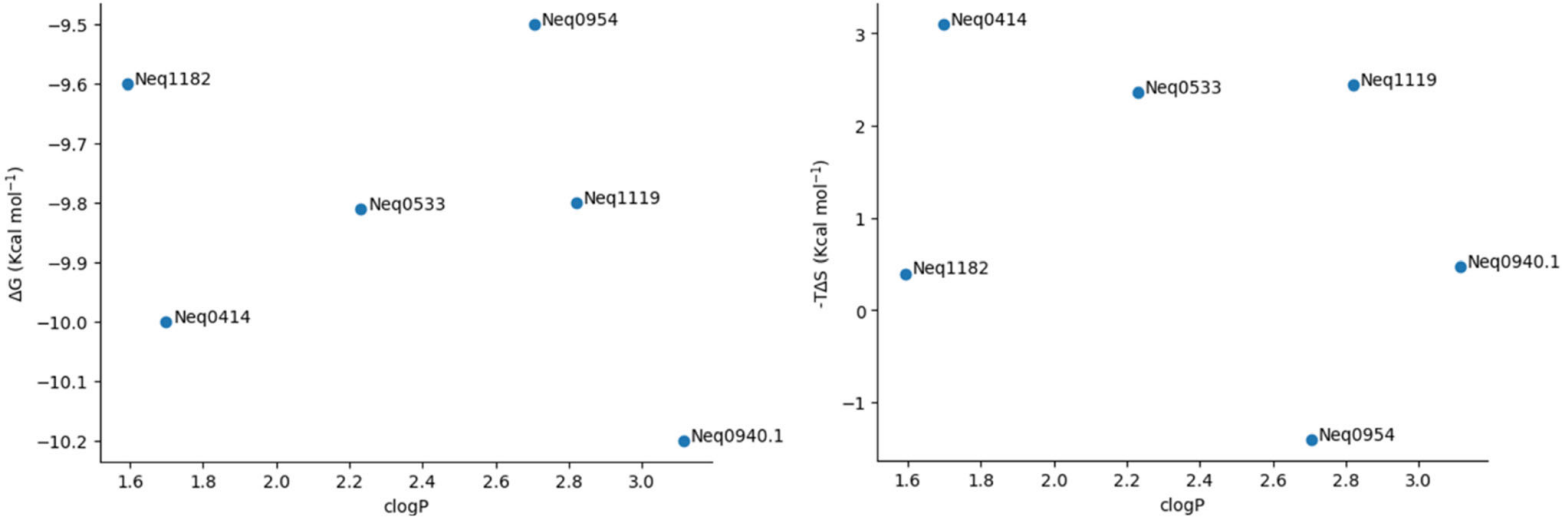
ΔG versus clogP plot (left) and –TΔS versus clogP plot (right) for the TBMP‐Phe‐P1‐CN series (six compounds).

**Figure 3:**
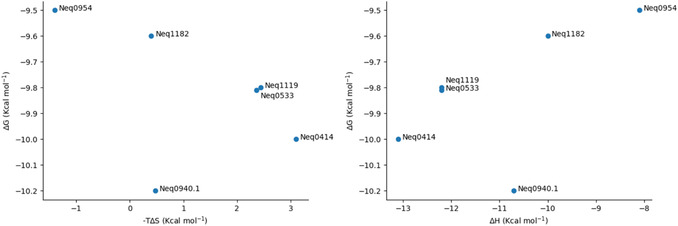
ΔG versus –TΔS plot (left) and ΔG versus ΔH plot (right) for the TBMP‐Phe‐P1‐CN series (six compounds).

To investigate the effect of lipophilicity on the free energy of binding, plots of ΔG versus clogP (**Figure** **2**, left) and –TΔS versus clogP (Figure 2, right) were created for the TBMP‐Phe‐P1‐CN series. Additionally, **Figure** **3** shows how individual components affect binding, with plots of ΔG versus –TΔS (left) and ΔG versus ΔH (right) for the same series.

The lipophilicity plots show that, for ΔG, data points are scattered with no clear pattern, while for –TΔS, there seems to be an expected descending pattern from Neq0414 to Neq0940.1, that is, more lipophilic compounds leading to less positive entropic contributions. However, both Neq0954 and Neq1182 deviate completely from the –TΔS trend, with Neq1182 being the most hydrophilic compound in the series, but with a near‐zero entropic contribution.

The plots of ΔG versus –TΔS (Figure 3, left) and ΔH (Figure 3, right) show that, except for one outlier (Neq0940.1), binding in the TBMP‐Phe‐P1‐CN series is mostly enthalpically driven, as the most potent compounds are also those with the more negative enthalpies of binding. These are, however, offset by the entropic factor, a consequence of the EEC phenomenon. According to Freire,^[^ ^65^ ^]^ with the evolution of a compound series, bulkier groups are added to scaffolds, which usually leads to compounds in which the enthalpic factor contributes more to the free energy of binding. The opposite is observed here since the enthalpy of binding is more negative for compounds with the smallest P1 moieties (Neq0414—methylene, Neq0533—cyclopropyl).

#### 4.5.2 TBMP‐Leu‐P1‐CN Series

**Figure 4:**
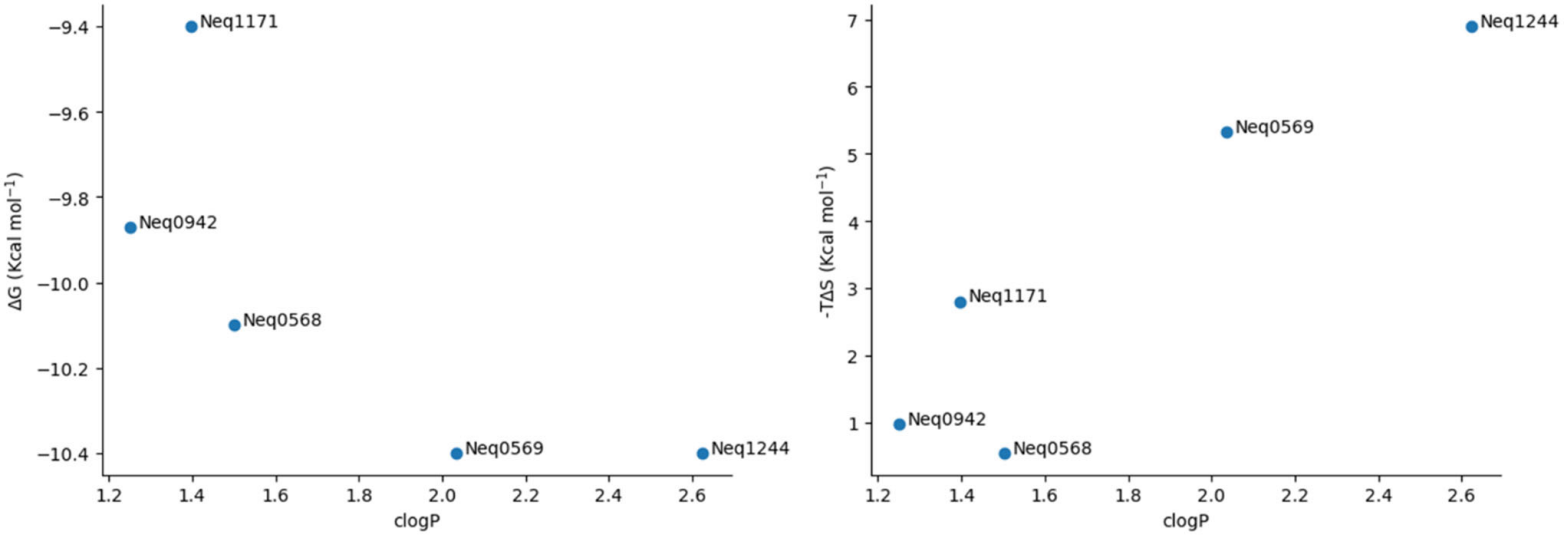
ΔG versus clogP plot (left) and –TΔS versus clogP plot (right) for the TBMP‐Leu‐P1‐CN series (five compounds).

**Figure 5:**
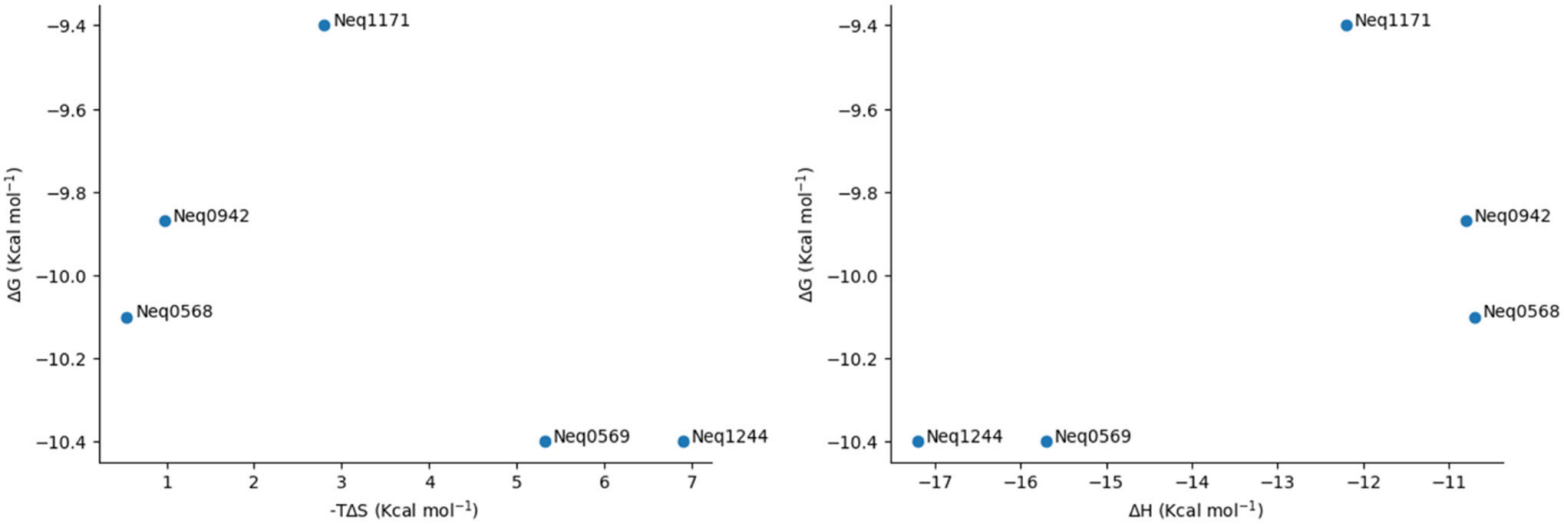
ΔG versus –TΔS plot (left) and ΔG versus ΔH plot (right) for the TBMP‐Leu‐P1‐CN series (five compounds).

**Figure** **4** shows the plots of ΔG versus clogP (left) and –TΔS versus clogP (right) for the TBMP‐Leu‐P1‐CN series, and **Figure** **5**, the plots of ΔG versus –TΔS (left) and ΔG versus ΔH (right) for the same series.

In this case, the ΔG versus clogP plot follows the expected pattern, with more lipophilic compounds leading to more negative ΔG, but Neq1171 presents itself as an outlier, with a ΔG less negative than the trend. The –TΔS versus clogP plot, on the other hand, is more unusual, with the more polar compounds Neq0568, Neq0942, and Neq1171 presenting lower entropic factors, while the more lipophilic Neq1244 shows a highly positive entropic factor. Neq1244 is not like Neq1119, its P2 = phenylalanine counterpart, since Neq1119 follows the expected trend of the series, while Neq1244 is, like Neq1171 and Neq1182, an outlier for entropy, being the compound with the highest lipophilicity in the series, but also the one with the largest positive entropic contribution.

**Figure 6:**
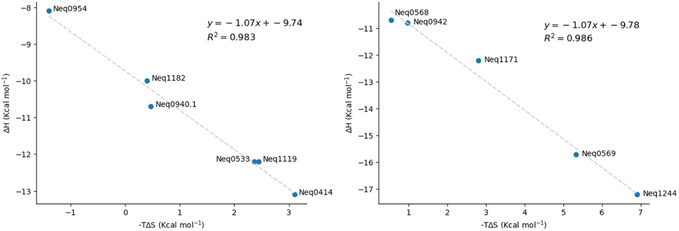
EEC phenomenon in both molecular series analyzed.

Unlike the previous series, binding in the TBMP‐Leu‐P1‐CN series does not seem to be either enthalpically or entropically driven. In both series, EEC plays a significant role, as shown in **Figure** **6**. Because of that, all compounds, regardless of P1 bulk or polarity, presented similar inhibition constants. Nevertheless, it is important to highlight that the inclusion of P1 moieties in cruzain inhibitors is not done exclusively to increase potency, and other effects, such as increasing selectivity against other enzymes and improving pharmacokinetic parameters, should also be considered when selecting a lead to develop.

In summary, the plots demonstrate that both series contain outliers, and once again indicate that lipophilicity is not the only factor impacting entropy of binding for this class of compounds. It is also interesting that even in flat regions of SAR (compounds with approximately the same free energy of binding), the entropic and enthalpic terms can be significantly different. Flat SARs presenting strong nonadditivities in enthalpy and entropy have been demonstrated and attributed to the effect of water networks.^[^ ^66^ ^,^ ^67^ ^]^ Therefore, harder to measure hydration effects must have a significant effect in this series, both for the more polar γ‐lactam derivatives Neq1171 and Neq1182 and for the methionine‐derivative Neq1244.

In another article from our group, the authors noted that most compounds showed detrimental entropic contributions and that the two compounds with favorable entropic contributions presented higher lipophilicity than the rest of the series. However, compounds from other classes were included in the SAR study, and a more careful analysis shows that one of those two compounds, 10 g, actually contains a polar pyridine group in P1, showing a favorable entropy of binding (–TΔS = –1.4 kcal mol^−1^) while preserving potency (*pK*_d_ = 7.0),^[^ ^28^ ^]^ which is in line with our results.

## 5 Conclusion

During the COVID‐19 pandemic the world saw a combined effort between academia, industry, and regulating agencies, which led to the discovery of several inhibitors of targets involved in the infection and brought nirmatrelvir (PAXLOVID, Pfizer) to market in record time. However, this process has also produced hundreds of compounds which will never reach the market for their original indications. In this work, we show that the chemical matter targeting this disease might contain inhibitors of cysteine proteases, including those relevant for neglected diseases, by using ML to repurpose a molecular series originally developed as SARS‐CoV‐2 inhibitors to target cruzain, the main cysteine protease of *T. cruzi*.

From a library of 52 compounds, 11 were selected by the model. Of these, three were not considered due to a lack of novelty, although they were expected to be cruzain inhibitors. Five of the remaining eight compounds were demonstrated to be active. Of those, four belonged to a matched molecular series and, along with three additional compounds, formed a molecular series selected for determination of thermodynamic fingerprints with ITC. With the ITC analysis, we observed an H/S anomaly, in that more polar analogs presented more favorable entropic contributions to binding than their more lipophilic partners. We hypothesized that a solvent effect would be driving this unexpected reversal but docking analyses with explicit water molecules were inconclusive.

Analysis of an extended molecular series demonstrated that some compounds were indeed outliers, but the relatively flat SARs with strong nonadditivities in entropy and enthalpy suggest the involvement of water network effects. More advanced methods, such as molecular dynamics simulations and NMR experiments have been used to explore the role of water in unexpected thermodynamic results,^[^ ^63^ ^]^ but these would be outside the scope of this work. It is also worth reminding that the limitations of ITC have been discussed,^[^ ^68^ ^]^ and that all interpretations of these results should be taken with caution, as it is not always possible to uncouple the highly complex phenomena that can impact the thermodynamics of binding. Finally, observing a low entropic contribution for a peptidomimetic compound that does not employ highly lipophilic moieties (Neq1182) is worth highlighting. This could be a starting point for improved inhibitors that “beat” the EEC, a prevalent phenomenon in all compounds studied in this work.

## Conflict of Interest

The authors declare no conflict of interest.

## Supporting information

Supplementary Material

## Data Availability

The data that support the findings of this study are available in the supplementary material of this article.
