# Supplementary material for: Machine Learning‐Guided Repositioning of a SARS‐CoV‐2‐Targeting Molecular Series as Cruzain Inhibitors
Source: ChemMedChem. 2026 Jan 31;21(2):e202500630. doi: 10.1002/cmdc.202500630 (PMC12860495; doi:10.1002/cmdc.202500630)
Supplement: Supplementary file 1 — Supplementary Material [file CMDC-21-e202500630-s001.pdf]

Disclaimer: Several of the compounds reported in this publication and their corresponding characterization data have been published in a patent:

Cysteine Proteases Inhibitor Compounds, Pharmaceutical Composition and Use of the Compounds in Preparation of a Medication for Treatment of Infections Caused by Coronavirus

Inventors: F. C. P. Martins, C. A. Montanari, A. Leitao, J. S. da Silva, V. Bonatto, A. Shamim

Publication date: 2024

Patent number: BR 10 2024 016304-4

Since the original characterization data is not available at the time of submission of this publication, it has been made available here.

Enzyme inhibition assays

1. Expression of cruzain

The plasmid pET-21a (Epoch Life Science) was introduced to *Escherichia coli* Arctic Express (DE3)RIL (Agilent Technologies) competent cells by thermal shock and incubated with SOC medium (Sigma-Aldrich) in a SIF600R Incubator Shaker (Lab. Companion) for 60 min and 250 rpm at 37 °C. An LB-agar plate with 100 µg/mL Ampicillin and 20 µg/mL Gentamicin was prepared to grow the transformed cells and incubated at 37 °C overnight. An isolated colony of bacteria was transferred to 25 g/L sterile LB medium containing the antibiotics and incubated in a shaker at 37 °C overnight (250 rpm). The optical density value was measured the next morning using an absorbance of 600 nm (ideal optical density value between 0.8 to 1.0) and the culture volume required to initiate optical density at 0.16 after transfer to 1 L of induction medium was calculated.

The induction sterile medium was prepared with 10 g/L N-Z-Amine (Sigma-Aldrich), 5 g/L yeast extract (Sigma-Aldrich), 0.5% v/v glycerol (Vetec), 0.045 M ammonium chloride (Vetec), 0.023 M potassium phosphate monobasic (Alfa-Aesar), 0.023 M dibasic sodium phosphate (Sigma-Aldrich), 0.0018 M magnesium sulfate (Sigma-Aldrich), 0.0045 M sodium sulfate (Sigma-Aldrich), 0.045% w/v D-glucose (Vetec), 0.18% w/v α-Lactose monohydrate (Sigma-Aldrich), 20 µg/mL Gentamicin (Sigma-Aldrich) and 100 µg/mL Ampicillin (Sigma-Aldrich). The calculated culture volume was then transferred to the induction medium, which was incubated in a shaker for 72 hours and 200 rpm at 18 °C. After finishing, the solution was centrifugated (Hitachi, model CR21GIII) for 20 min, 9000 rpm at 4 °C and the pellet obtained was stored at -80 °C until the purification step.

2. Purification of cruzain

The pellet was thawed, resuspended in 50 mL of lysis buffer consisting of 0.5 mM calcium chloride (Sigma-Aldrich), 0.5 mM magnesium sulfate (Sigma-Aldrich), 10 µL DNase (Promega) and a small amount of lysozyme (Sigma-Aldrich), and incubated in an ice bath for 60 min. Afterwards, the resuspension was sonicated for 5 minutes with 30 seconds “pulse on” and 30 seconds “pulse off” in a sonic dismembrator (Fisher Scientific Sonic Dismembrator, model 500). The solution was centrifuged for 30 min at 4 °C and 9000 rpm (Hitachi Centrifuge, model CR21GIII). The supernatant was mixed with 5 mL of nickel resin (Ni, Sepharose 6 fast flow, GE Healthcare) and left under agitation for 3 hours on a magnetic stirrer at 4 °C in a cold chamber.

The mixture was transferred to a bench column and was washed using buffer A pH 10.0 (50 mM Tris (Sigma-Aldrich), 300 mM calcium chloride (Synth) and 10 mM imidazole (Sigma-Aldrich)). The protein was eluted in buffer B pH 10.0 (50 mM Tris (Sigma-Aldrich), 300 mM calcium chloride (Synth) and 500 mM imidazole (Sigma-Aldrich)) and was transferred to a 10 kDa dialysis membrane (Dialysis tubing cellulose, Sigma-Aldrich) that was submerged in activation buffer pH 5.5 at 4 °C under overnight agitation. The activation buffer was previously prepared containing

100 mM sodium acetate (Sigma-Aldrich), 5 mM EDTA (Sigma-Aldrich), and 300 mM sodium chloride (Synth). Next day, the activation buffer was exchanged, and the protein remained under agitation for another 3 hours. When finished, the pH was adjusted (pH = 5.0 if protein precipitation occurred, otherwise, pH = 5.2) and diluted to a concentration equal to or less than 0.5 mg/mL to prevent protein precipitation during the activation step.

3. Activation of cruzain

β-mercaptoethanol (Sigma-Aldrich) was added to the diluted protein solution from the purification step at a final concentration 1 mM – it acts as a cysteine reducing agent. The solution was incubated at 37 °C for 1 hour and 80 rpm. During this process, the solution went from cloudy to clear as the enzyme lost its N-terminal prodomain. After the activation, the protein solution was concentrated using an Amicon® Ultra 10 kDa membrane (Merck Millipore) at 4 °C for 30 min and 4600 rpm. The concentrated protein solution was aliquoted into separate 500 µL fractions, immediately frozen in liquid nitrogen and stored at -80 °C.

4. Determination of the Michaelis constant (K_M)

The enzymatic kinetics assays were performed in a fluorimeter equipment (Biotek Synergy HT), using black 96-well microplates (Corning®) at 37 °C. The excitation wavelength used was 360 nm and emission was measured at 460 nm. The enzymes were activated in 100 mM sodium acetate buffer pH 5.5 (Sigma-Aldrich), 300 mM sodium chloride (Synth) and 5 mM EDTA (Sigma-Aldrich), containing 7 mM DTT (Sigma-Aldrich), and 0.014% Triton X-100 (Sigma-Aldrich). The activation solution was incubated in an ice bath for 10 min. The enzyme concentration used was 0.15 nM and a final concentration of 5% of DMSO (Sigma-Aldrich) was used in each well. The fluorogenic substrate used was Z-Phe-Arg-7-amido-4-methylcoumarin (Z-FR-MCA, Sigma-Aldrich) in eight different concentrations which were prepared with a 0.5 dilution factor. The reaction rate was followed by 5 min and the hydrolysis rate of the fluorogenic substrate was measured by the Gen5™ Biotek software. The assay was performed in triplicates.

5. Determination of inhibition constants (K_i)

The enzymatic activity was evaluated by fluorimetric assays (Biotek Synergy HT) monitoring the hydrolysis rate of the fluorogenic substrate Z-Phe-Arg-7-amido-4-methylcoumarin (Z-FR-MCA, Sigma-Aldrich) with fluorescence emission at 460 nm and excitation at 355 nm. Enzyme kinetic assays were carried out in Corning 96-well black flat bottom microplates containing 200 µL of a solution constituted by 100 mM sodium acetate buffer pH 5.5 (Sigma-Aldrich), 300 mM sodium chloride (Synth), 5 mM EDTA (Sigma-Aldrich), 5 mM DTT (dithiothreitol), 5% v/v DMSO (dimethyl sulfoxide), 0.01% v/v Triton X-100 (Sigma-Aldrich) and 0.15 nM cruzain. First, the enzyme stock aliquot was thawed and incubated in an ice bath for 15 min using the activation buffer. Then, the reaction was followed over 5 min at 37 °C, of which 2 min with the measured inhibitors, before adding the substrate to trigger the reaction.

Visual inspection and a pre-reading of plate wells were performed to check for possible precipitation and background fluorescence, respectively. None of the substances displayed a significant fluorescence signal around 460 nm, the emission wavelength used to monitor reaction kinetics. Thus, potential inner-filter effects did not have to be considered in our experiments. Analysis and manipulation of the data were performed with Sigma Plot 10.

The experiments were performed in triplicate for each inhibitor. Initial velocities of substrate hydrolysis under the first-order reaction were calculated using Gen5™ Biotek software. The apparent inhibition constant K_i' was determined by non-linear regression using Equation 1:

$$V_S = \frac{V_0}{1 + \frac{[I]}{K_i'}} \quad \text{Eq. 1}$$

where V_S is the steady-state rate, V_0 is the rate in the absence of inhibitor, and [I] is the inhibitor concentration. The true inhibition constant K_i was calculated by the correction of K_i' according to Equation 2:

$$K_i = \frac{K_i'}{1 + \frac{[S]}{K_M}} \quad \text{Eq. 2}$$

where [S] is the substrate concentration and K_M is the Michaelis constant. The concentration of the substrate used in the assays was fixed and kept equal to the previously calculated value of K_M ([S] = K_M), and all inhibitors were evaluated at seven different concentrations. The initial concentration is 10 µM (which is adjusted to 1 µM in case the inhibitor behaves as a strong inhibitor), which is then sequentially diluted at a 1:1 ratio for a total of 7 dilutions. Strong inhibitors have a higher affinity and saturate the enzyme's active sites more quickly, directly interfering with the calculation of the inhibition constant, which makes it necessary to use a lower initial concentration. A control measurement with the covalent reversible fast-binding inhibitor Neq0570 (SMILES: O=C(C1=CC=CC=C1)N[C@H](C(NC2(C#N)CC2)=O)CC3=CC=CC=C3) was performed for each setup plate.

Synthesis and characterization

Amide synthesis

To a solution of the carboxylic acid (1.2 mmol, 1.2 equiv.), HATU (1.2 mmol, 1.2 equiv.), and DIPEA (0.45 mL, 2.6 mmol, 2.6 equiv.) in anhydrous DMF (5 mL), the free primary amine (1.0 mmol, 1.0 equiv.) was added under an argon atmosphere in a round bottom flask. The resulting mixture was stirred at room temperature for 20 h. After completion, the reaction was diluted with ethyl acetate (10 mL) and washed with saturated aqueous $NaHCO_3$ (3 × 20 mL) and saturated aqueous NaCl (3 × 20 mL). The organic layer was dried over anhydrous Na_2SO_4, concentrated under reduced pressure, and purified by flash column chromatography.

BOC deprotection

The BOC-protected compound (1.0 mmol, 1.0 equiv.) was dissolved in anhydrous CH_2Cl_2 (3 mL), and trifluoroacetic acid (TFA) (0.91 mL, 8.0 mmol, 9.0 equiv.) was added dropwise at 0 °C. The reaction mixture was stirred at room temperature for 2 h and monitored by TLC. The solvent and excess TFA were removed under reduced pressure to afford the crude product, which was used in the next step without further purification.

Ester hydrolysis to carboxylic acid

The ester was dissolved in THF (30 mL) and treated with aqueous LiOH (2 M, 30 mL). The reaction mixture was stirred at room temperature for 2 h and monitored by TLC. After completion, the solution was acidified with 2 M HCl to pH 1, and extracted with ethyl acetate (5×). The organic phase was dried over anhydrous Na_2SO_4 and concentrated under reduced pressure to yield the corresponding carboxylic acid.

Conversion of ester to amide

Method A: The ester was dissolved in 25% aqueous NH_4OH (4.3 mL) and methanol (4.3 mL), and the mixture was stirred until complete consumption of the starting material (monitored by TLC). The solvent was removed under reduced pressure, and the residue was extracted with ethyl acetate, water, and saturated NaCl solution (3×). The product was used in the next step without further purification.

Method B: A solution of ammonia in methanol (54.7 mmol, 54.7 equiv.) was added to the ester (1.0 mmol, 1.0 equiv.) and the mixture was stirred at 25 °C for 48 h. The solvent was removed under reduced pressure and to afford the crude product, which was used in the next step without further purification.

Dehydration of primary amide to nitrile

The primary amide (1.0 mmol, 1.0 equiv.) was dissolved in anhydrous CH_2Cl_2 (5 mL) under an argon atmosphere. Burgess reagent (3.5 mmol, 3.5 equiv.) was added in portions over 2 h while stirring. After the addition was complete, the reaction was stirred for an additional 15 min (monitored by TLC). The solvent was removed under reduced pressure, and the crude product was purified by flash column chromatography.

Reduction of ester to alcohol

A solution of the ester in methanol was cooled to 0 °C, and $NaBH_4$ was added in portions. The reaction mixture was stirred at room temperature for 2 h until complete consumption of the starting material (monitored by TLC). The mixture was then quenched with water and extracted with ethyl acetate. The organic phase was washed with brine, dried over anhydrous Na_2SO_4, filtered, and concentrated under reduced pressure. The crude product was purified by flash column chromatography.

Oxidation with Dess–Martin Periodinane (DMP)

To a solution of the alcohol (1.0 mmol, 1.0 equiv.) in anhydrous CH_2Cl_2 (10 mL) at 0 °C, Dess–Martin periodinane (1.2 mmol, 1.2 equiv.) was added. The reaction mixture was stirred at 0 °C for 30 min and then at room temperature for an additional 60 min. Upon completion, the mixture was quenched with saturated aqueous $NaHCO_3$ and diluted with ethyl acetate. The organic phase was washed with water and brine, dried over anhydrous Na_2SO_4, filtered, and concentrated under reduced pressure. The crude product was purified by flash column chromatography on silica gel.

Oxime formation from aldehydes

The aldehyde (1.0 mmol, 1.0 equiv.) and hydroxylamine hydrochloride (2.0 mmol, 2.0 equiv.) were dissolved in ethanol (5 mL), and the reaction mixture was stirred at room temperature for 12 h. After completion, the solvent was removed under reduced pressure. The residue was diluted with ethyl acetate (20 mL) and washed with water (2 × 10 mL). The organic layer was dried over anhydrous Na_2SO_4, filtered, and concentrated under reduced pressure. The crude product was purified by flash column chromatography.

Characterization

Neq1119

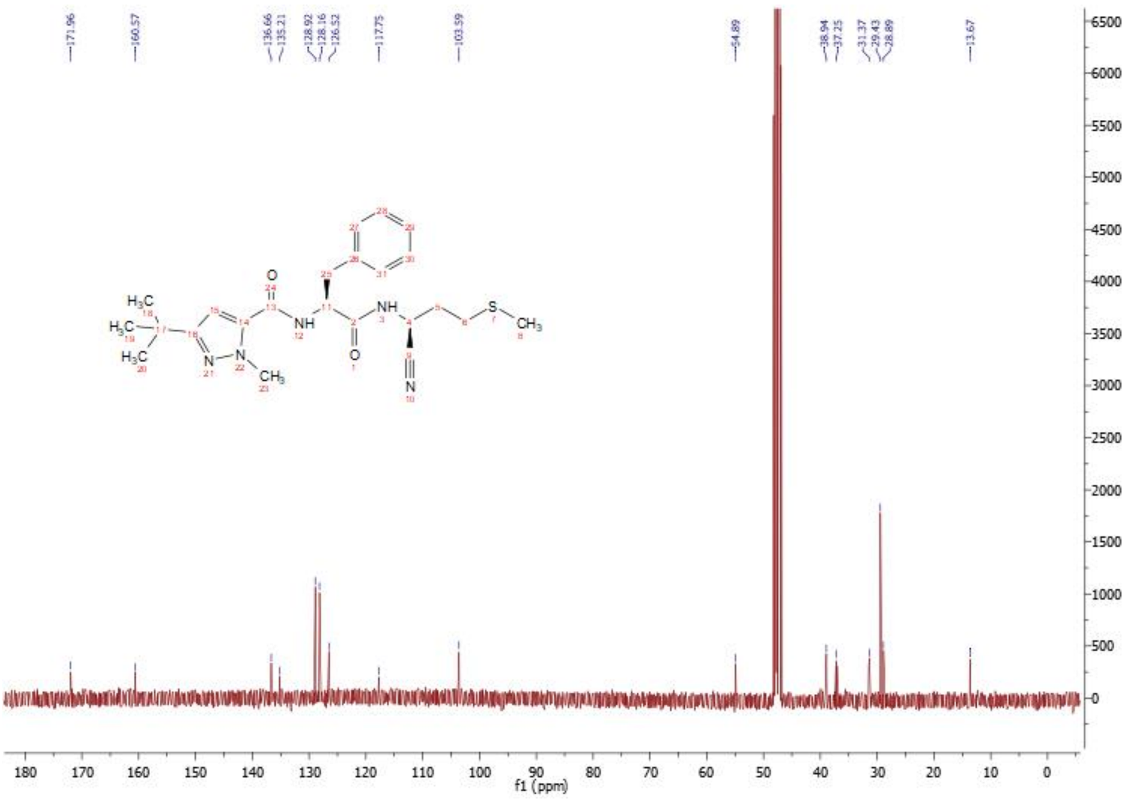

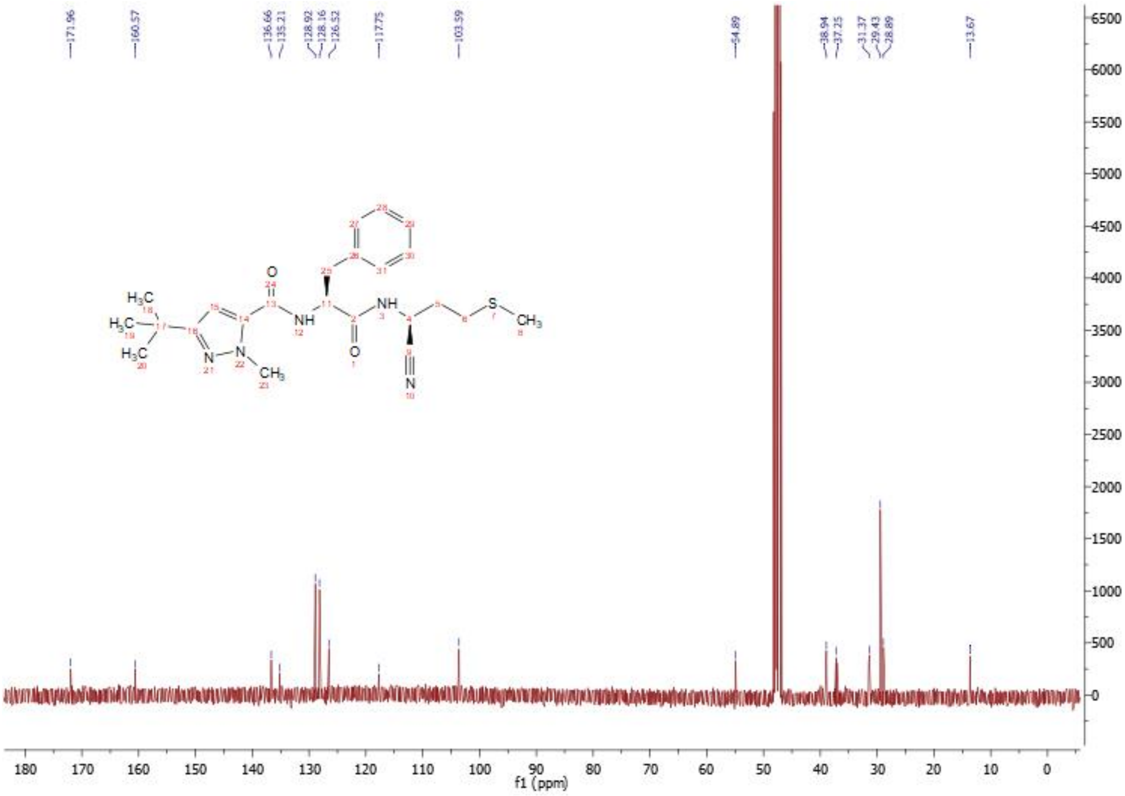

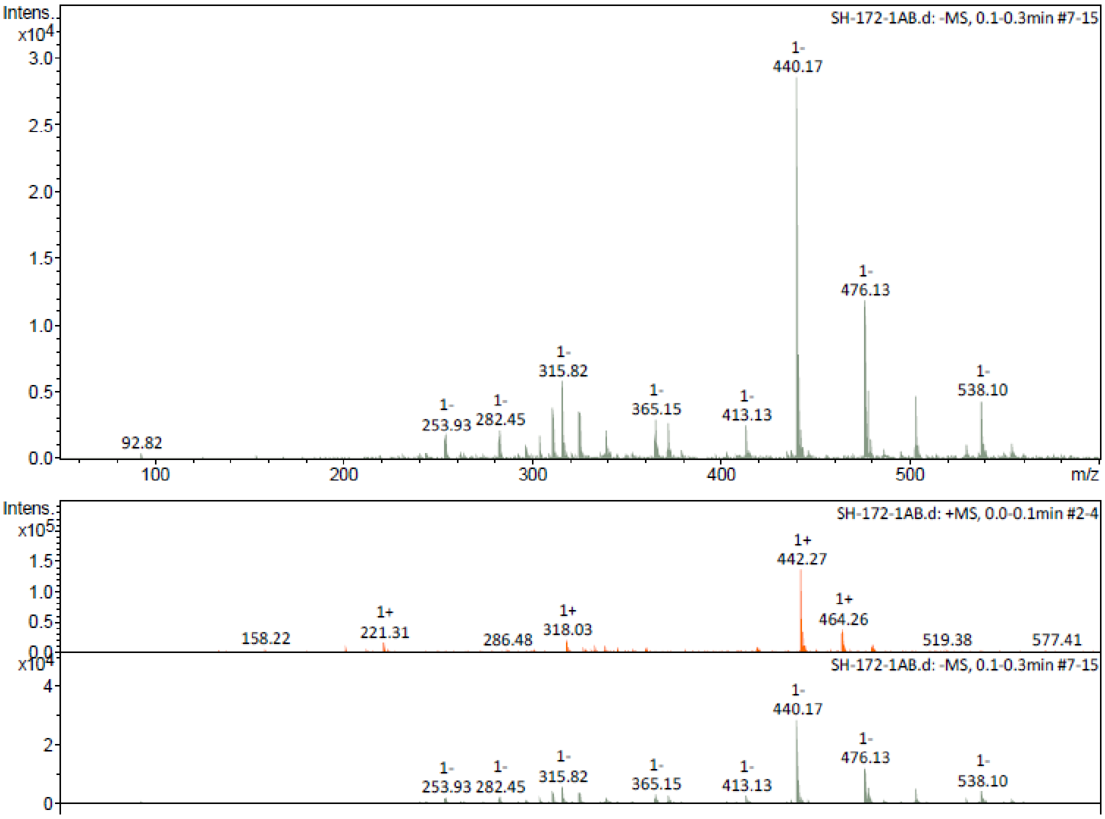
SH-172-1AB.d: -MS, 0.1-0.3min #7-15
Intens. x10^4
m/z
440.17
476.13
538.10
413.13
365.15
315.82
282.45
253.93
92.82
SH-172-1AB.d: +MS, 0.0-0.1min #2-4
Intens. x10^5
442.27
464.26
519.38
577.41
318.03
286.48
221.31
158.22

Neq1122

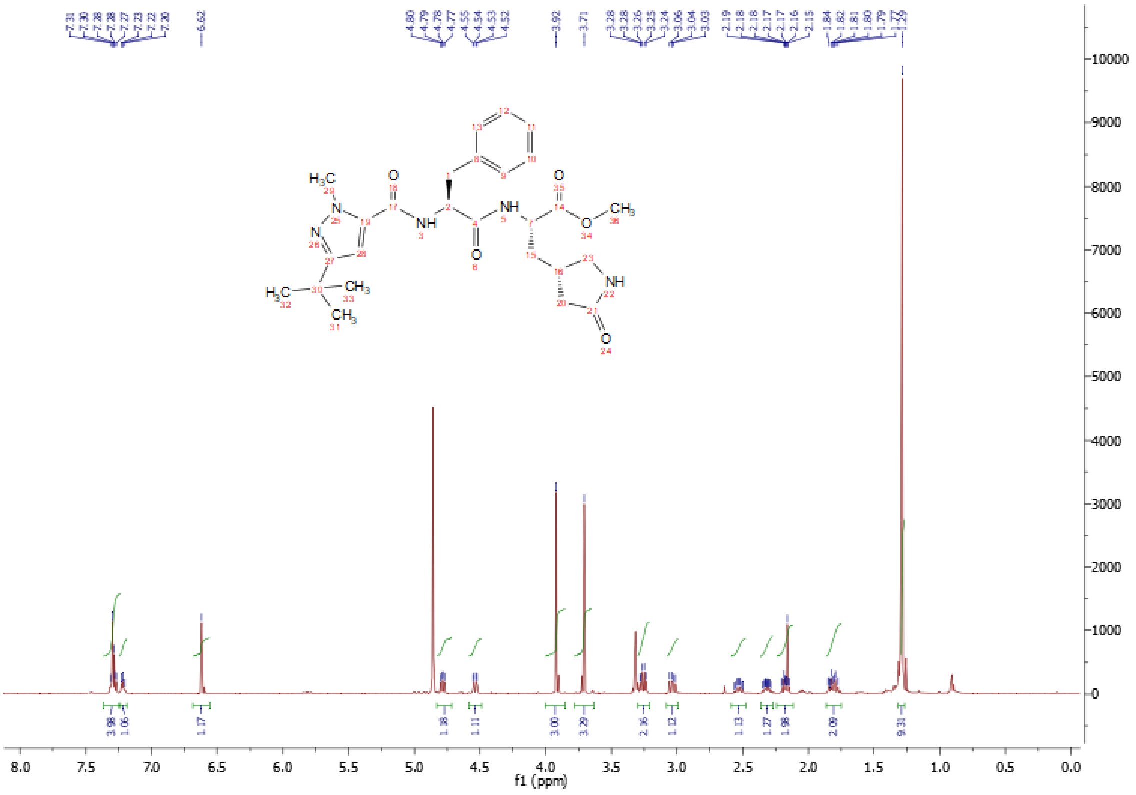
f1 (ppm)

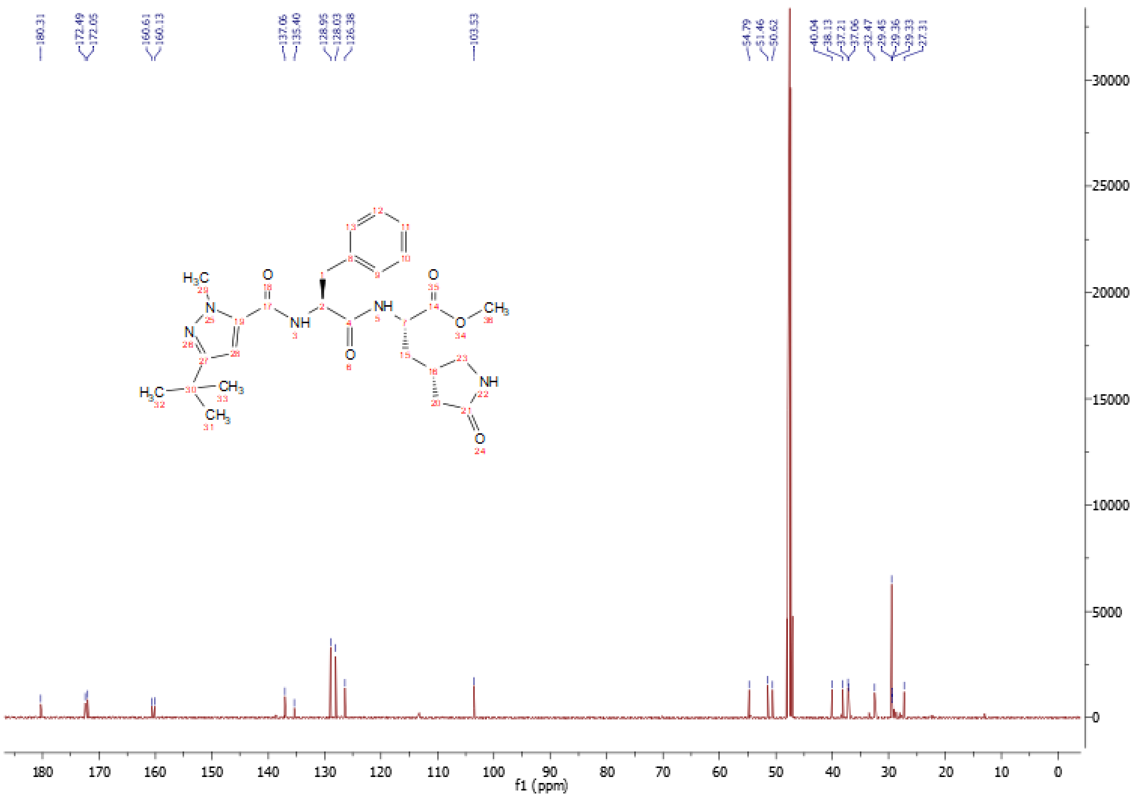
180.31
172.49
172.05
160.61
160.13
137.06
135.40
128.95
128.03
126.38
103.53
54.79
51.46
50.62
40.04
38.13
37.21
37.06
32.47
29.45
29.36
29.33
27.31
f1 (ppm)

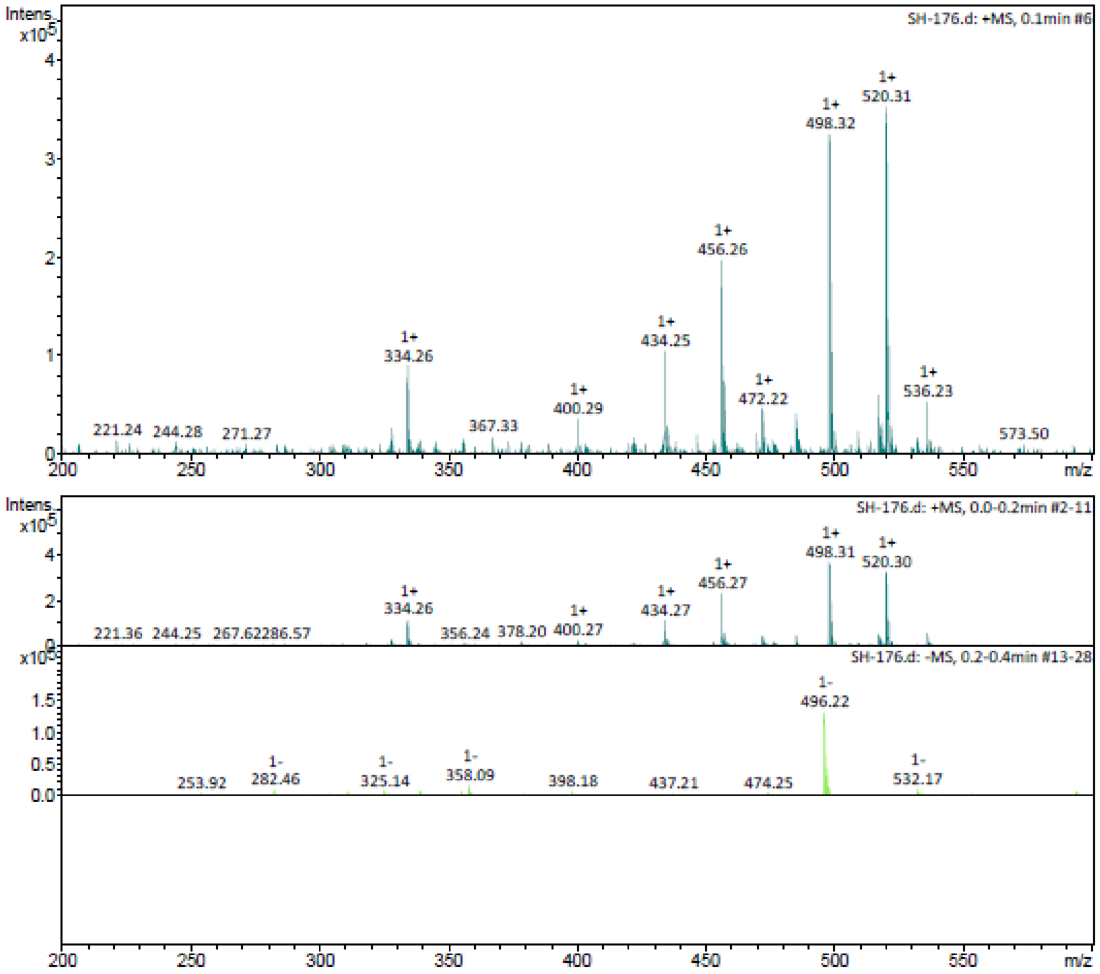
SH-176.d: +MS, 0.1min #6
Intens.
x10^5
1+ 520.31
1+ 498.32
1+ 456.26
1+ 434.25
1+ 334.26
1+ 400.29
1+ 472.22
1+ 536.23
221.24
244.28
271.27
367.33
573.50
SH-176.d: +MS, 0.0-0.2min #2-11
1+ 498.31
1+ 520.30
1+ 456.27
1+ 434.27
1+ 334.26
1+ 400.27
221.36
244.25
267.62286.57
356.24
378.20
SH-176.d: -MS, 0.2-0.4min #13-28
1- 496.22
1- 282.46
1- 325.14
1- 358.09
1- 532.17
253.92
398.18
437.21
474.25
m/z

Neq1133

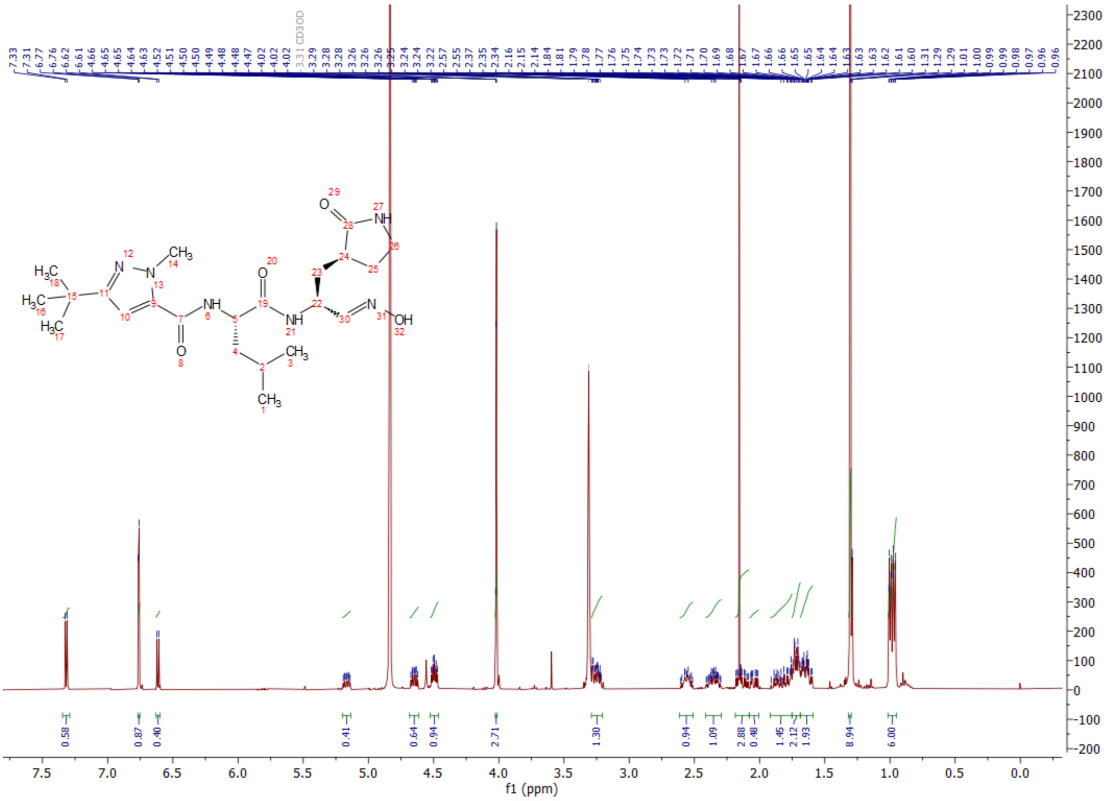

7.33 7.31 6.77 6.76 6.62 6.61 4.66 4.66 4.65 4.65 4.64 4.63 4.52 4.51 4.50 4.50 4.49 4.48 4.48 4.47 4.02 4.02 4.02 4.02 3.31 CD3OD 3.29 3.28 3.28 3.26 3.26 3.25 3.24 3.24 3.22 2.57 2.55 2.37 2.35 2.34 2.16 2.15 2.14 1.84 1.81 1.79 1.78 1.77 1.76 1.75 1.74 1.73 1.73 1.72 1.71 1.70 1.69 1.68 1.67 1.67 1.66 1.66 1.65 1.65 1.64 1.64 1.63 1.63 1.63 1.62 1.61 1.60 1.31 1.29 1.29 1.01 1.00 0.99 0.99 0.98 0.97 0.96 0.96
0.58 0.87 0.40 0.41 0.64 0.94 2.71 1.30 0.94 1.09 2.88 0.48 1.45 2.12 1.93 8.94 6.00
f1 (ppm)

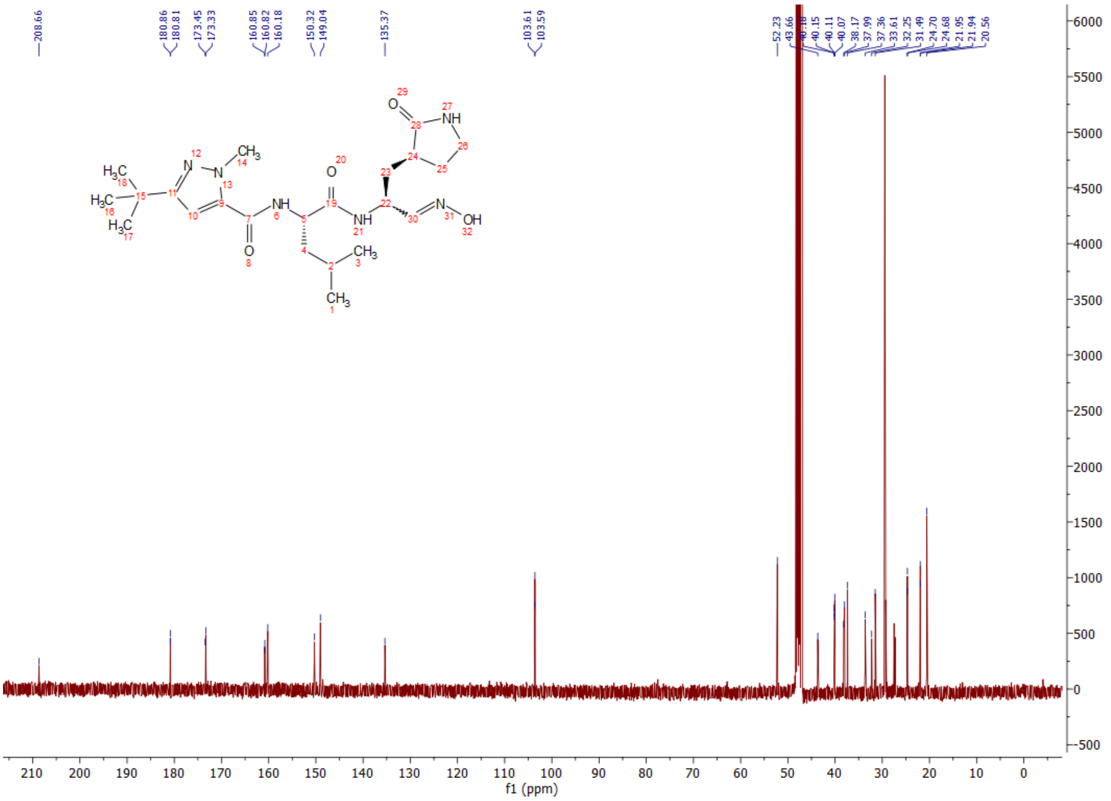

208.66 180.86 180.81 173.45 173.33 160.85 160.82 160.18 150.32 149.04 135.37 103.61 103.59 52.23 43.66 40.15 40.11 40.07 38.17 37.99 37.36 33.61 32.25 31.49 24.70 24.68 21.95 21.94 20.56
f1 (ppm)

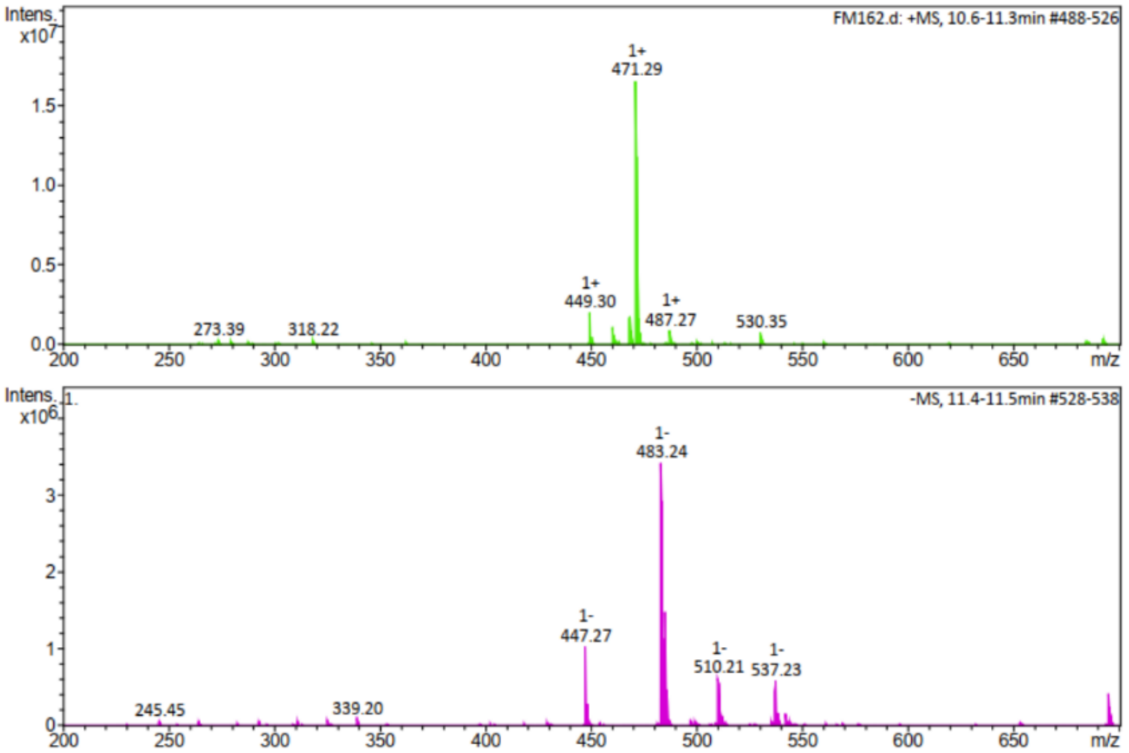

Intens. x10^7
FM162.d: +MS, 10.6-11.3min #488-526
1+ 471.29
1+ 449.30
1+ 487.27
530.35
273.39
318.22
m/z
Intens. x10^6
-MS, 11.4-11.5min #528-538
1- 483.24
1- 447.27
1- 510.21
1- 537.23
245.45
339.20
m/z

Neq1134

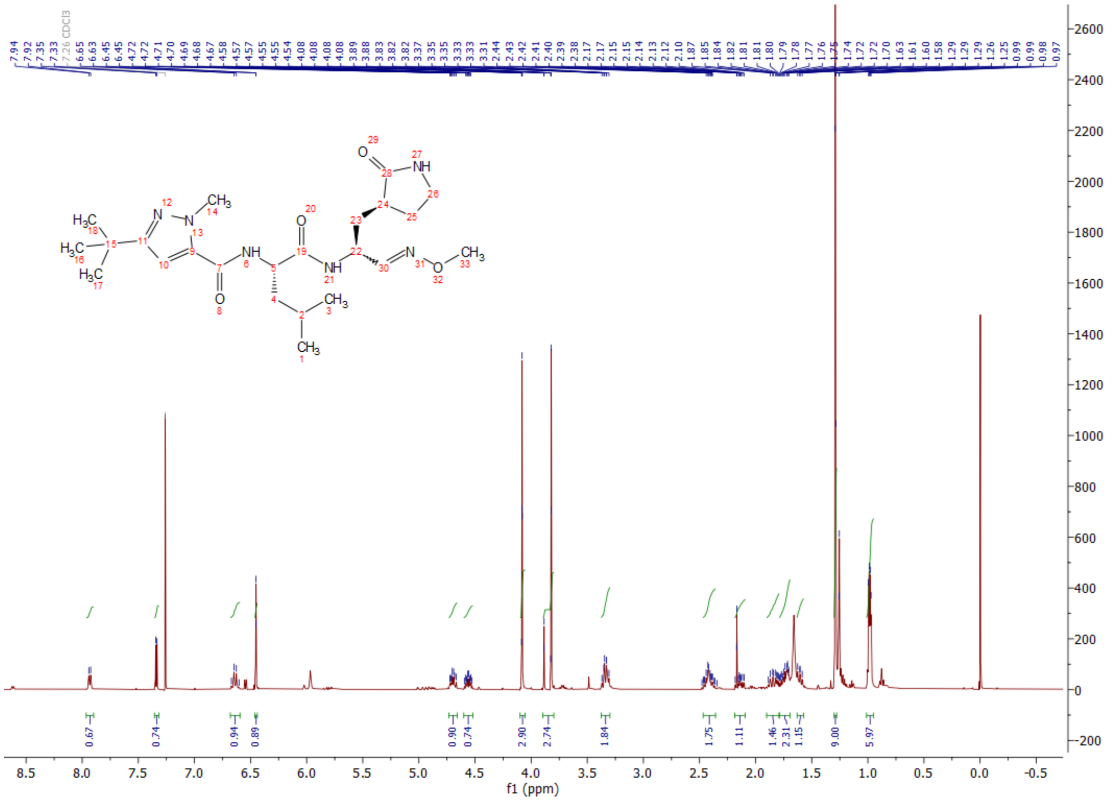

7.26 CDCl3
H_3C
CH_3
NH
O
N
f1 (ppm)
0.67
0.74
0.94
0.89
0.90
0.74
2.90
2.74
1.84
1.75
1.11
1.46
2.31
1.15
9.00
5.97

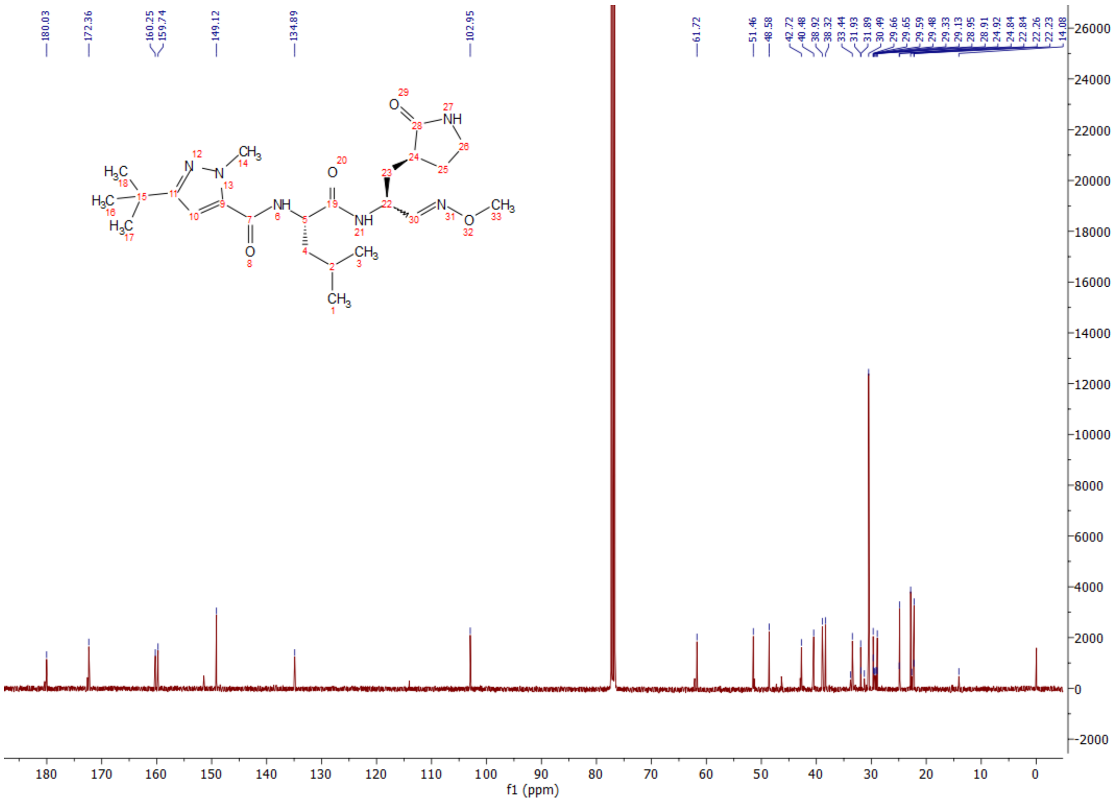
180.03
172.36
160.25
159.74
149.12
134.89
102.95
61.72
51.46
48.58
f1 (ppm)

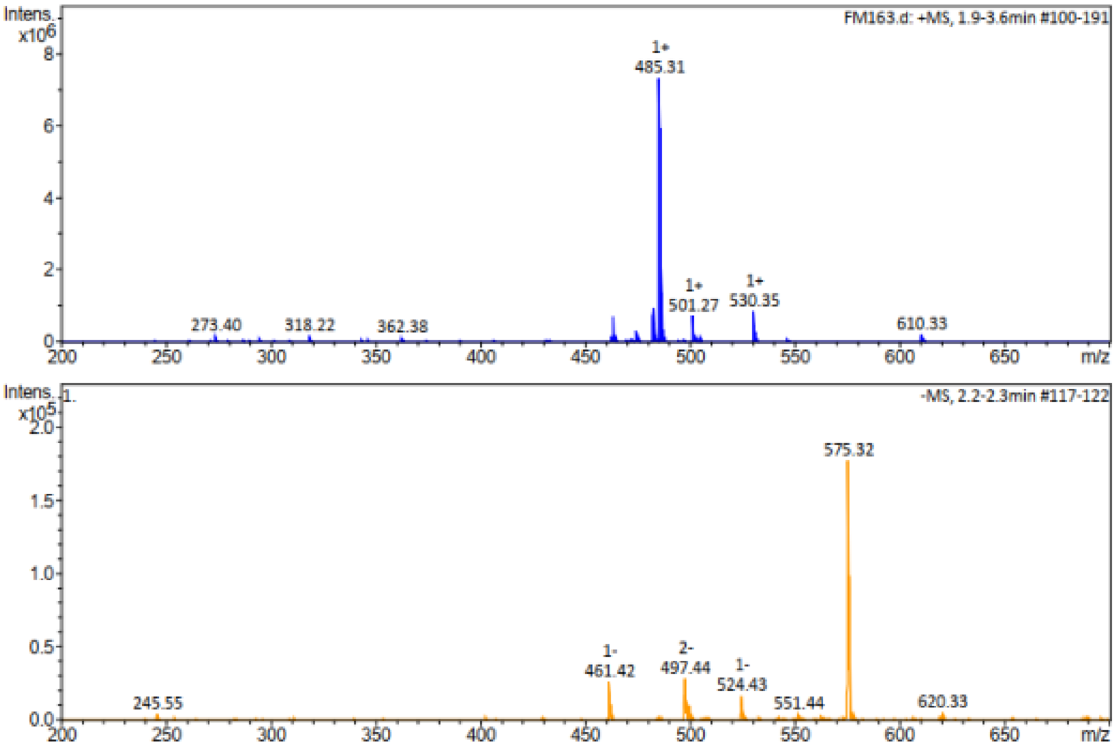
Intens.
x10^6
FM163.d: +MS, 1.9-3.6min #100-191
1+
485.31
1+
501.27
1+
530.35
273.40
318.22
362.38
610.33
m/z
Intens.
x10^5
-MS, 2.2-2.3min #117-122
575.32
1-
461.42
2-
497.44
1-
524.43
245.55
551.44
620.33
m/z

Neq1137

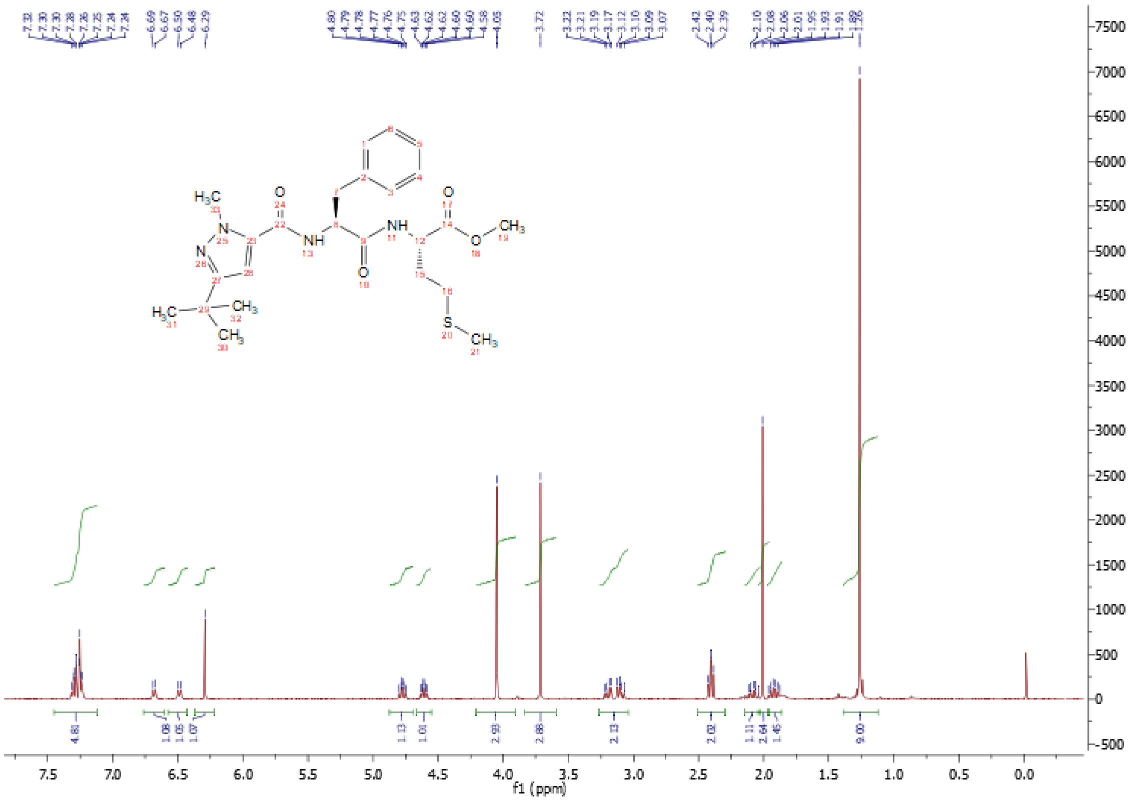
7.32 7.30 7.30 7.28 7.26 7.25 7.24 7.24
6.69 6.67 6.50 6.48 6.29
4.80 4.79 4.78 4.77 4.76 4.75 4.63 4.62 4.62 4.60 4.60 4.58 4.05
3.72
3.22 3.21 3.19 3.17 3.12 3.10 3.09 3.07
2.42 2.40 2.39
2.10 2.08 2.06 2.01 1.95 1.93 1.91 1.89 1.26
4.81 1.08 1.05 1.07 1.13 1.01 2.93 2.88 2.13 2.02 1.11 2.64 1.45 9.00
f1 (ppm)

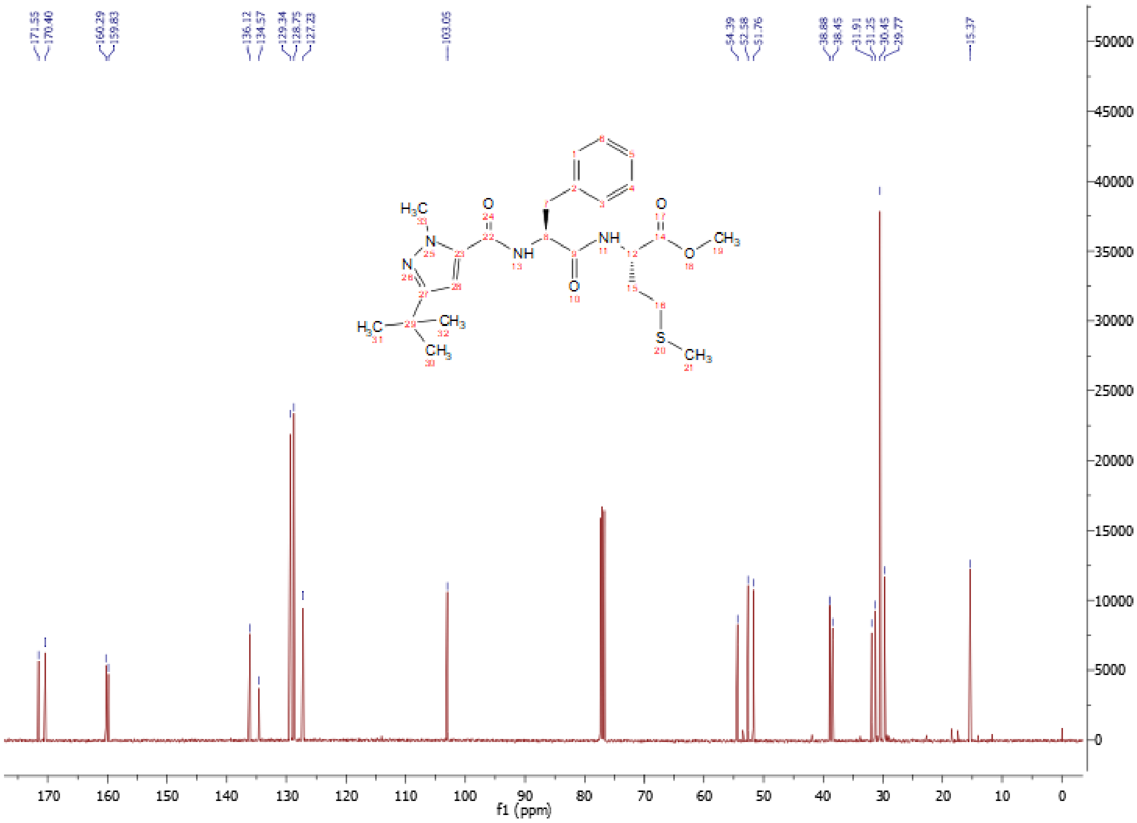
171.55 170.40 160.29 159.83 136.12 134.57 129.34 128.75 127.23 103.05 54.39 52.58 51.76 38.88 38.45 31.91 31.25 30.45 29.77 15.37
f1 (ppm)

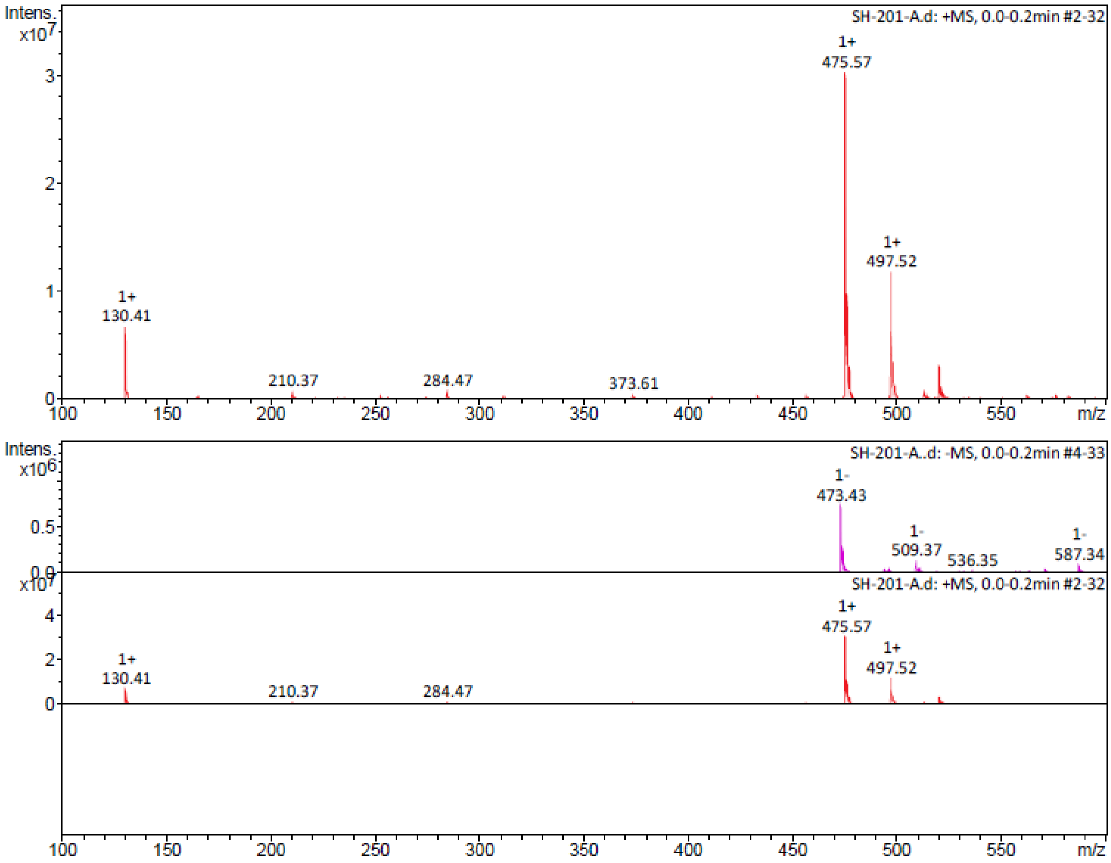
SH-201-A.d: +MS, 0.0-0.2min #2-32
SH-201-A..d: -MS, 0.0-0.2min #4-33
Intens. x10^7
475.57
497.52
130.41
210.37
284.47
373.61
473.43
509.37
536.35
587.34
m/z

Neq1148

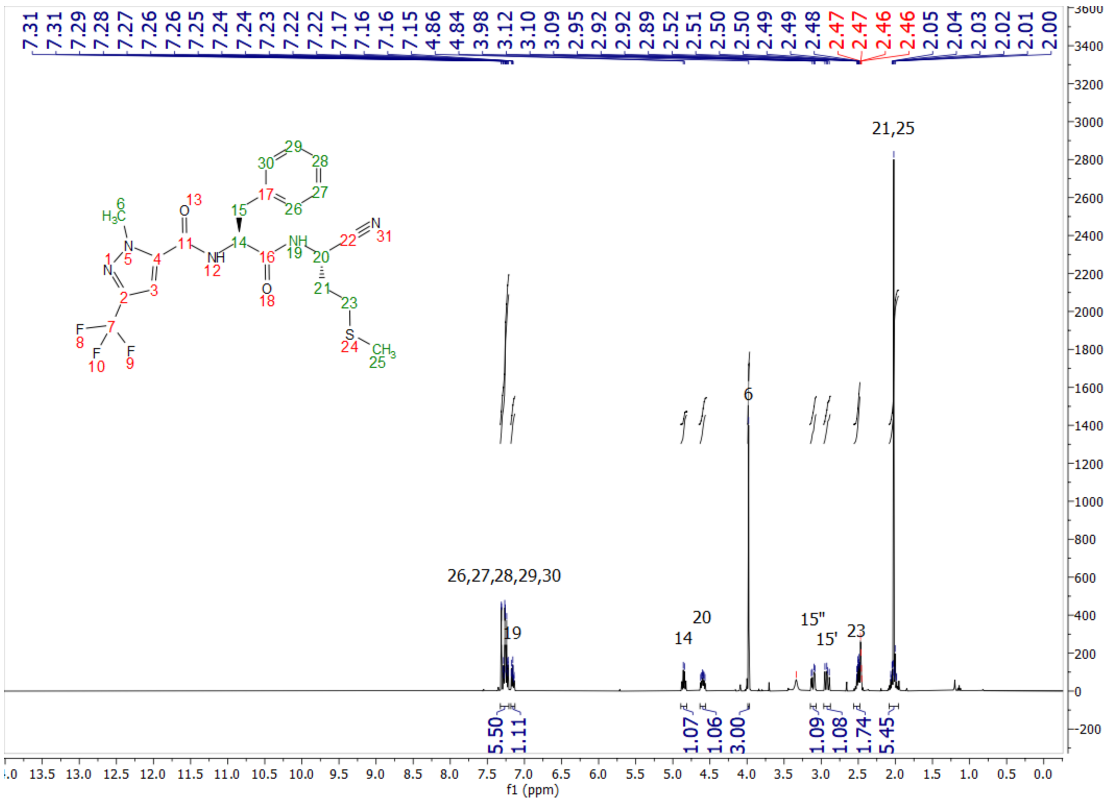
21,25
26,27,28,29,30
19
14
20
6
15"
15'
23
f1 (ppm)

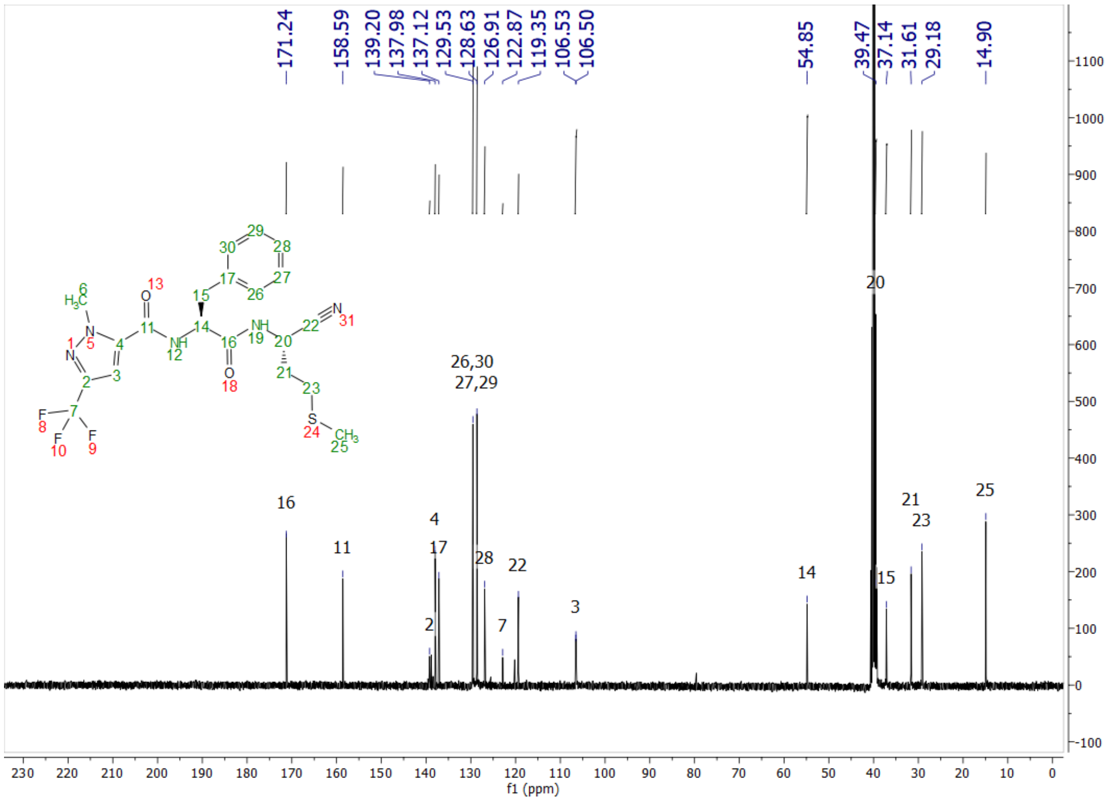

171.24
158.59
139.20
137.98
137.12
129.53
128.63
126.91
122.87
119.35
106.53
106.50
54.85
39.47
37.14
31.61
29.18
14.90
f1 (ppm)

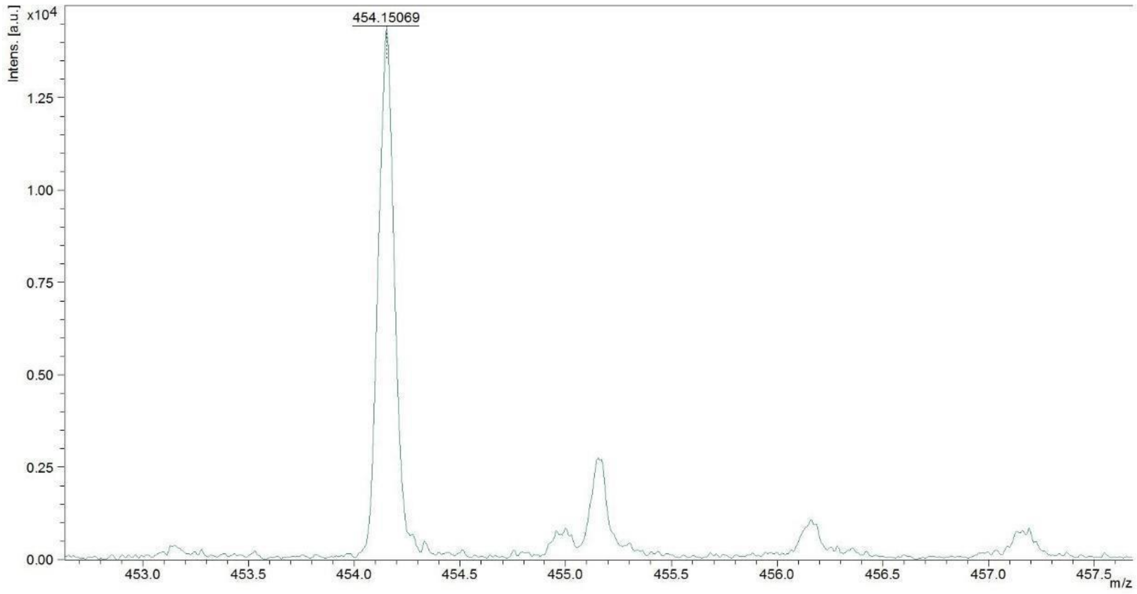

Intens. [a.u.]
x10^4
454.15069
m/z

Neq1171

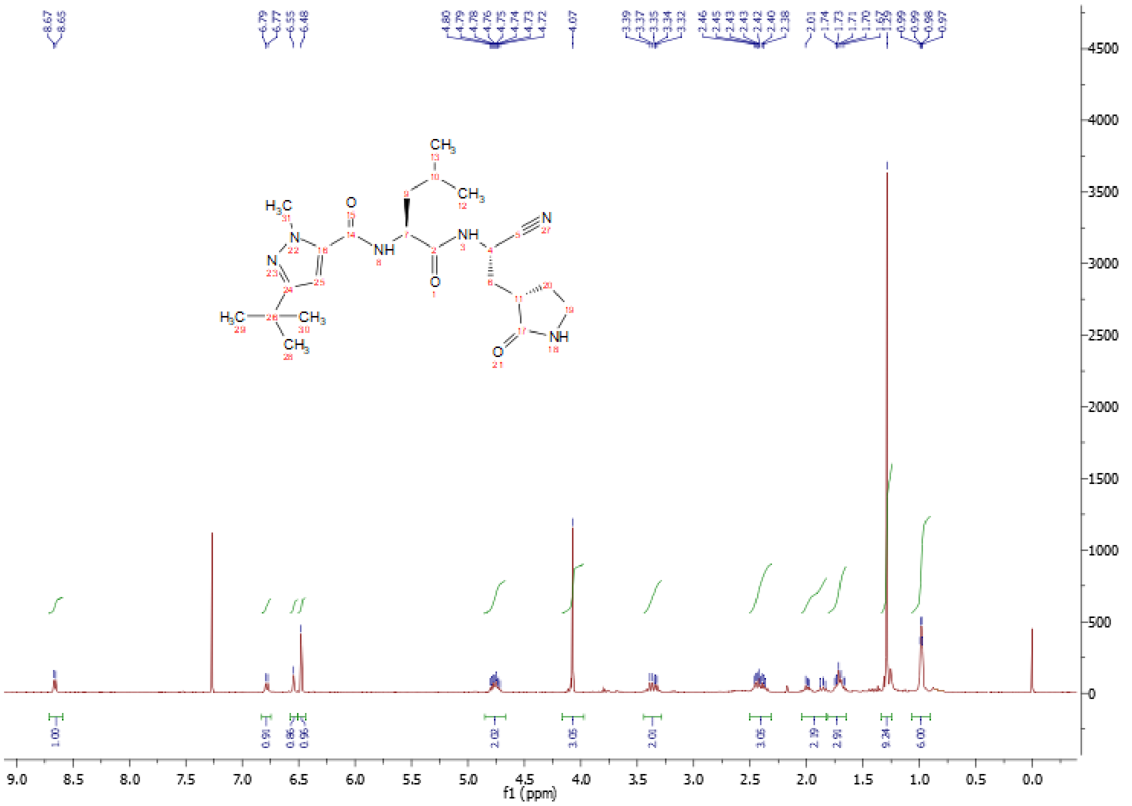

8.67
8.65
6.79
6.77
6.55
6.48
4.80
4.79
4.78
4.76
4.75
4.74
4.73
4.72
4.07
3.39
3.37
3.35
3.34
3.32
2.46
2.45
2.43
2.43
2.42
2.40
2.38
2.01
1.74
1.73
1.71
1.70
1.67
1.29
0.99
0.99
0.98
0.97
1.00
0.91
0.86
0.96
2.02
3.05
2.01
3.05
2.19
2.91
9.24
6.00
f1 (ppm)

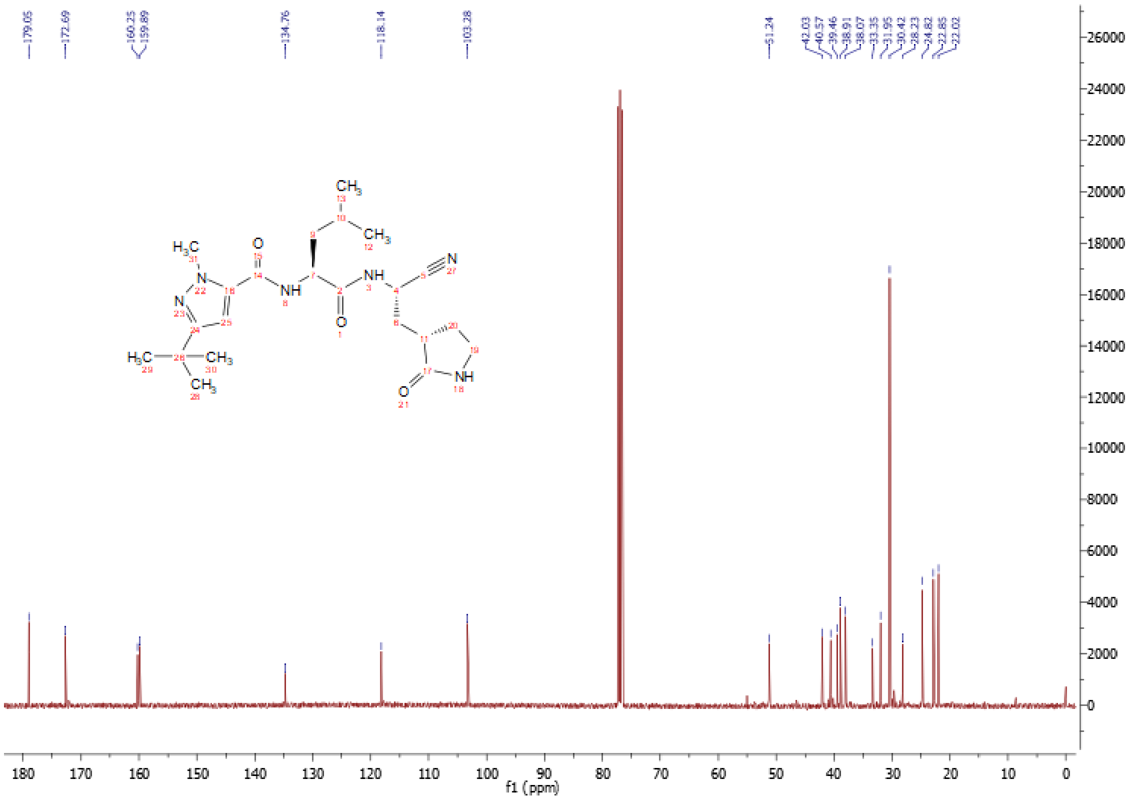

179.05
172.69
160.25
159.89
134.76
118.14
103.28
51.24
42.03
40.57
39.46
38.91
38.07
33.35
31.95
30.42
28.23
24.82
22.85
22.02
f1 (ppm)

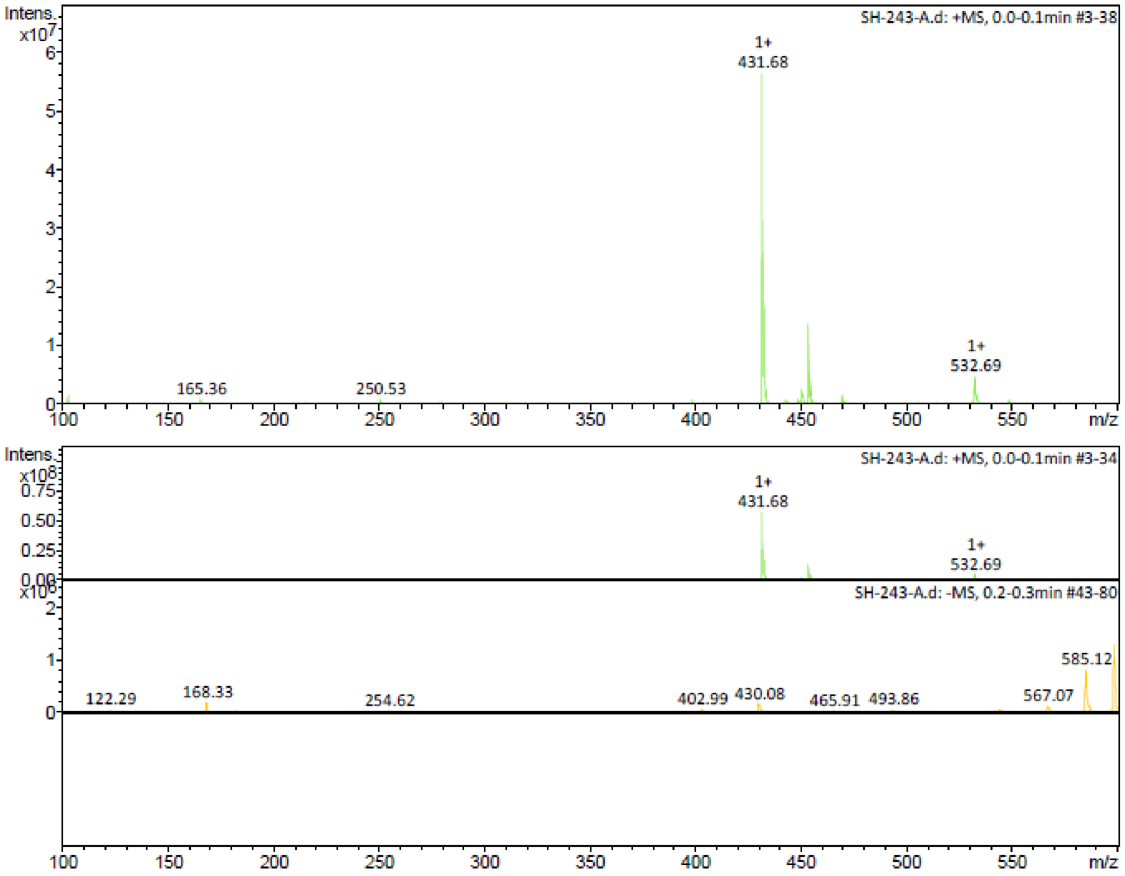

SH-243-A.d: +MS, 0.0-0.1min #3-38
SH-243-A.d: +MS, 0.0-0.1min #3-34
SH-243-A.d: -MS, 0.2-0.3min #43-80
Intens.
m/z

Neq1172

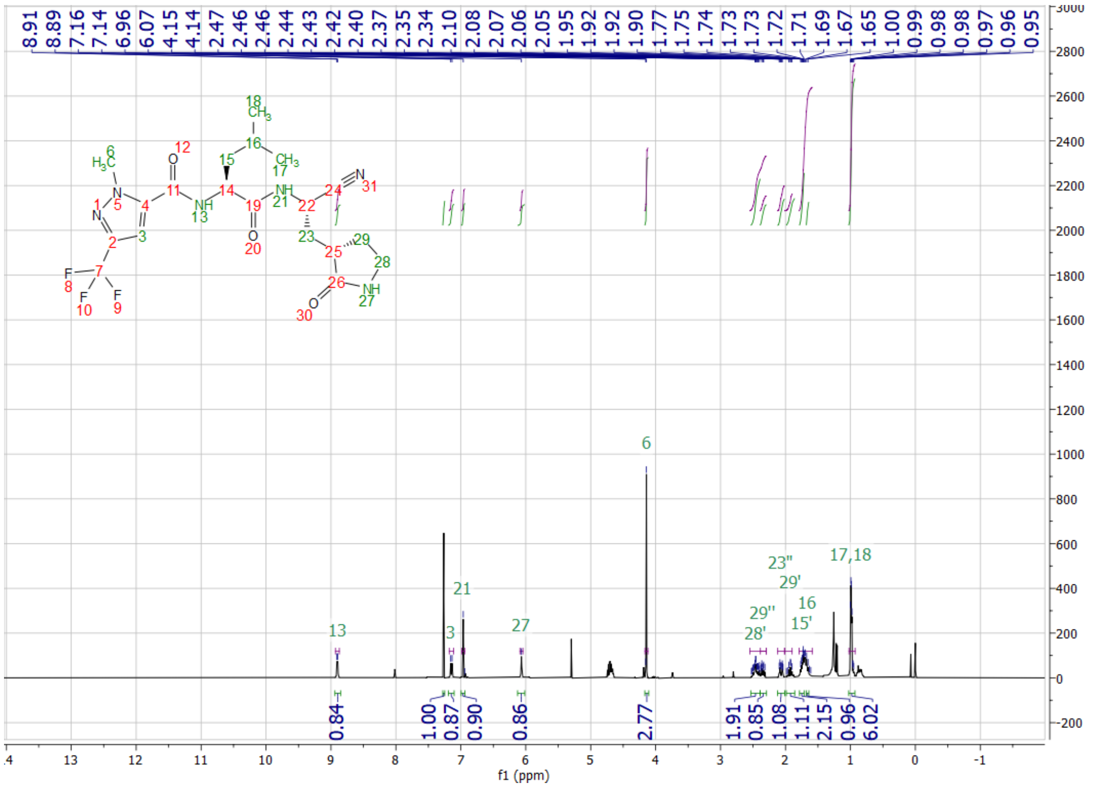

f1 (ppm)

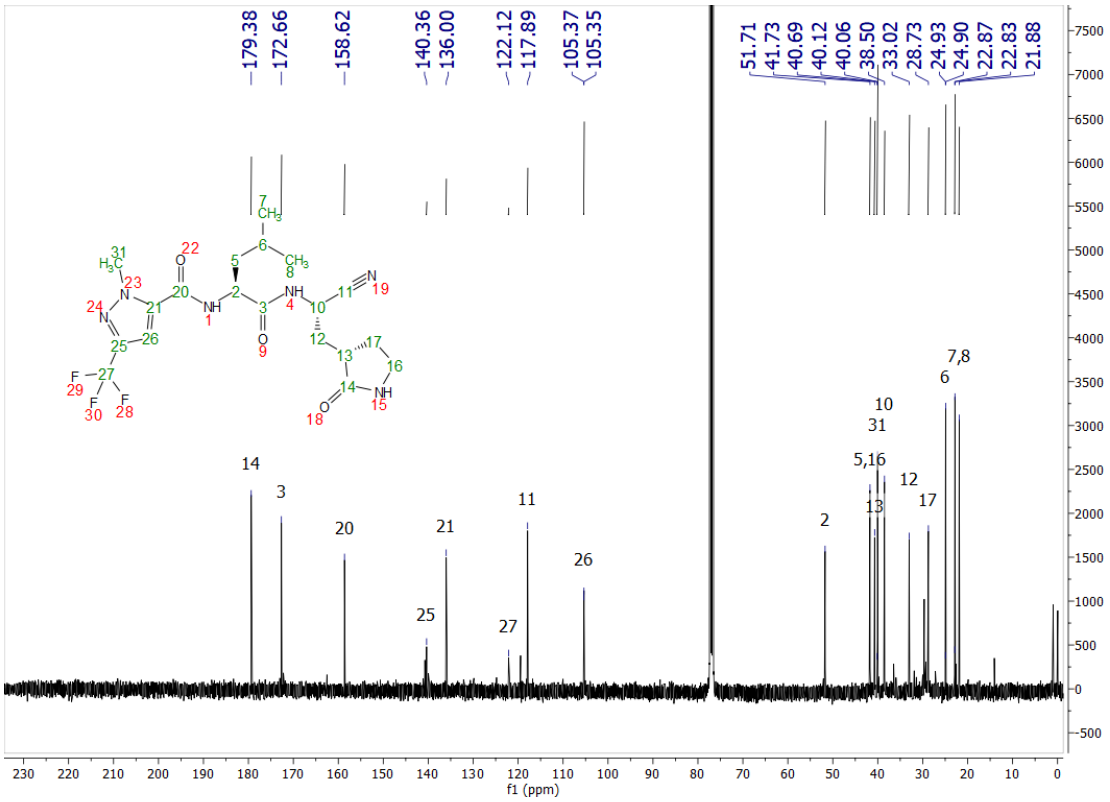
179.38
172.66
158.62
140.36
136.00
122.12
117.89
105.37
105.35
51.71
41.73
40.69
40.12
40.06
38.50
33.02
28.73
24.93
24.90
22.87
22.83
21.88
14
3
20
25
21
27
11
26
2
5,16
13
10
31
12
17
6
7,8
f1 (ppm)

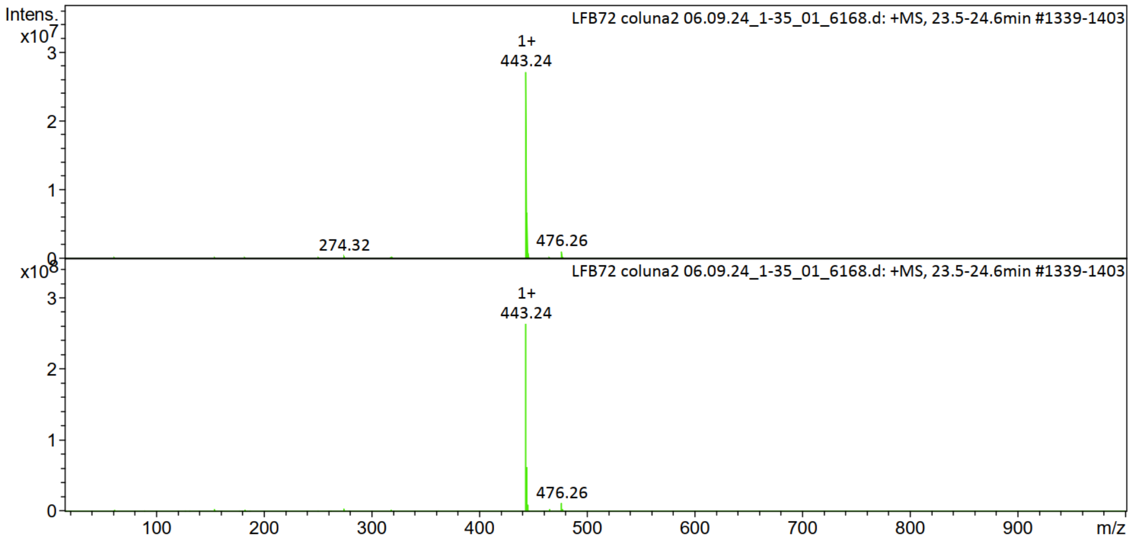
Intens.
x10^7
LFB72 coluna2 06.09.24_1-35_01_6168.d: +MS, 23.5-24.6min #1339-1403
1+
443.24
274.32
476.26
x10^8
LFB72 coluna2 06.09.24_1-35_01_6168.d: +MS, 23.5-24.6min #1339-1403
1+
443.24
476.26
m/z

Neq1182

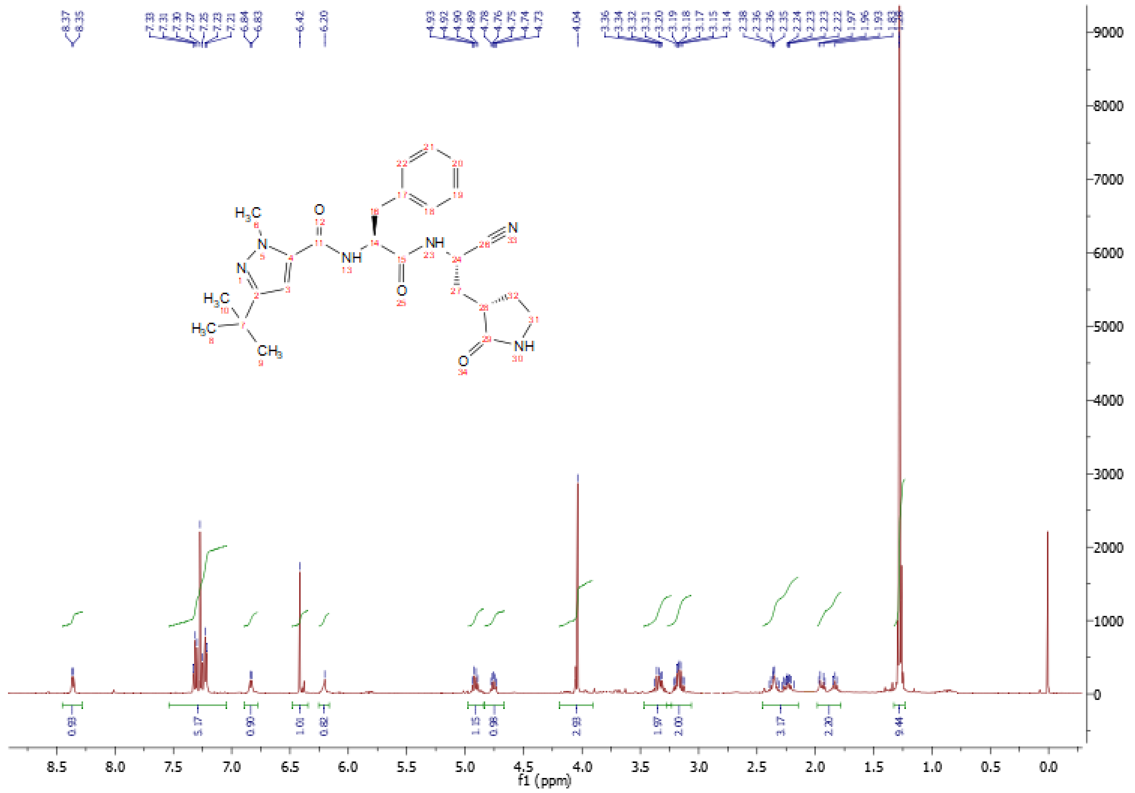

8.37 8.35 7.33 7.31 7.30 7.27 7.25 7.23 7.21 6.84 6.83 6.42 6.20 4.93 4.92 4.90 4.89 4.78 4.76 4.75 4.74 4.73 4.04 3.36 3.34 3.32 3.31 3.20 3.19 3.18 3.17 3.15 3.14 2.38 2.36 2.36 2.35 2.24 2.23 2.23 2.22 1.97 1.96 1.93 1.83
0.93 5.17 0.90 1.01 0.82 1.15 0.98 2.93 1.97 2.00 3.17 2.20 9.44
f1 (ppm)

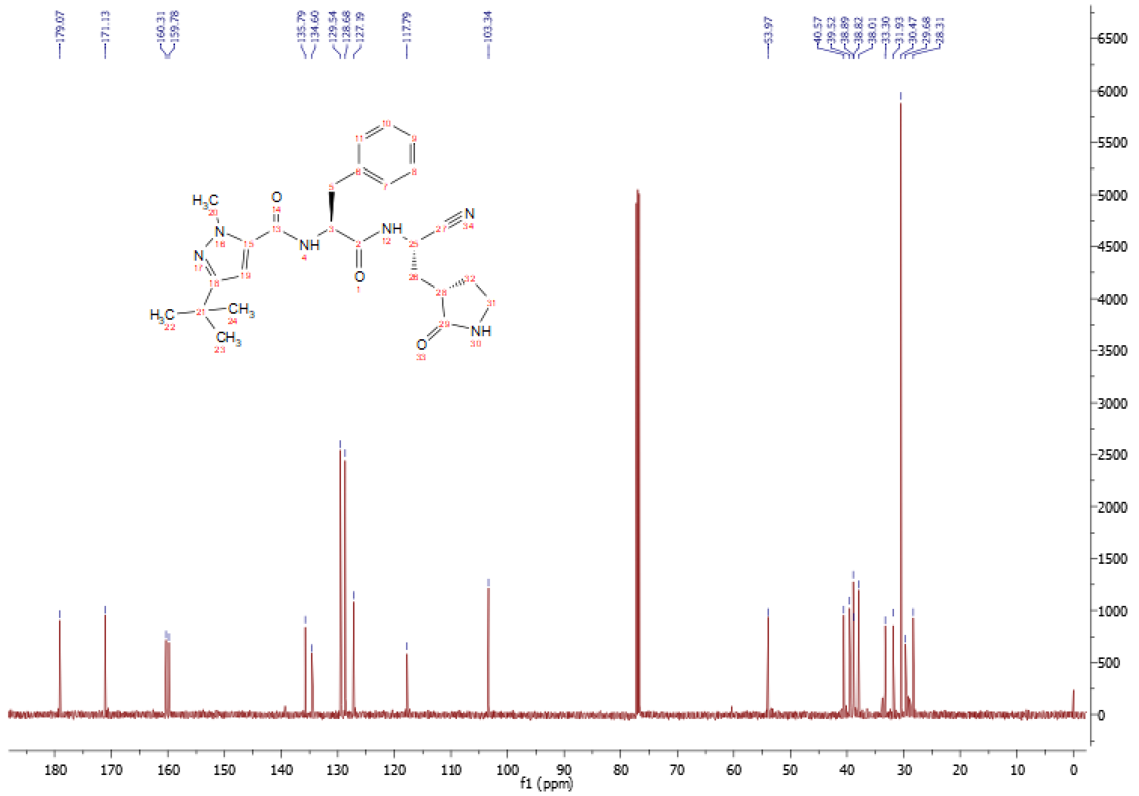

179.07 171.13 160.31 159.78 135.79 134.60 129.54 128.68 127.39 117.79 103.34 53.97 40.57 39.52 38.89 38.82 38.01 33.30 31.93 30.47 29.68 28.31
f1 (ppm)

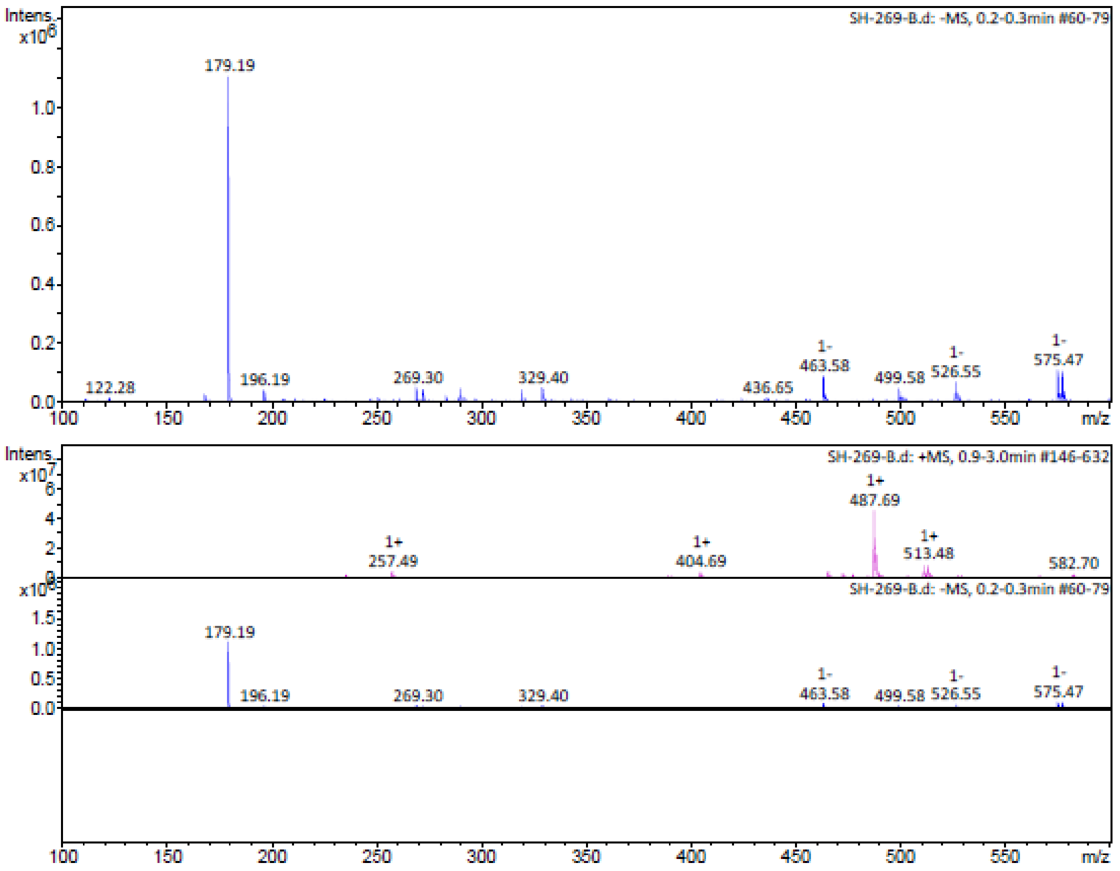
Intens.
x10^6
SH-269-B.d: -MS, 0.2-0.3min #60-79
179.19
122.28
196.19
269.30
329.40
436.65
1-
463.58
499.58
1-
526.55
1-
575.47
m/z
SH-269-B.d: +MS, 0.9-3.0min #146-632
1+
487.69
1+
257.49
1+
404.69
1+
513.48
582.70

Neq1244

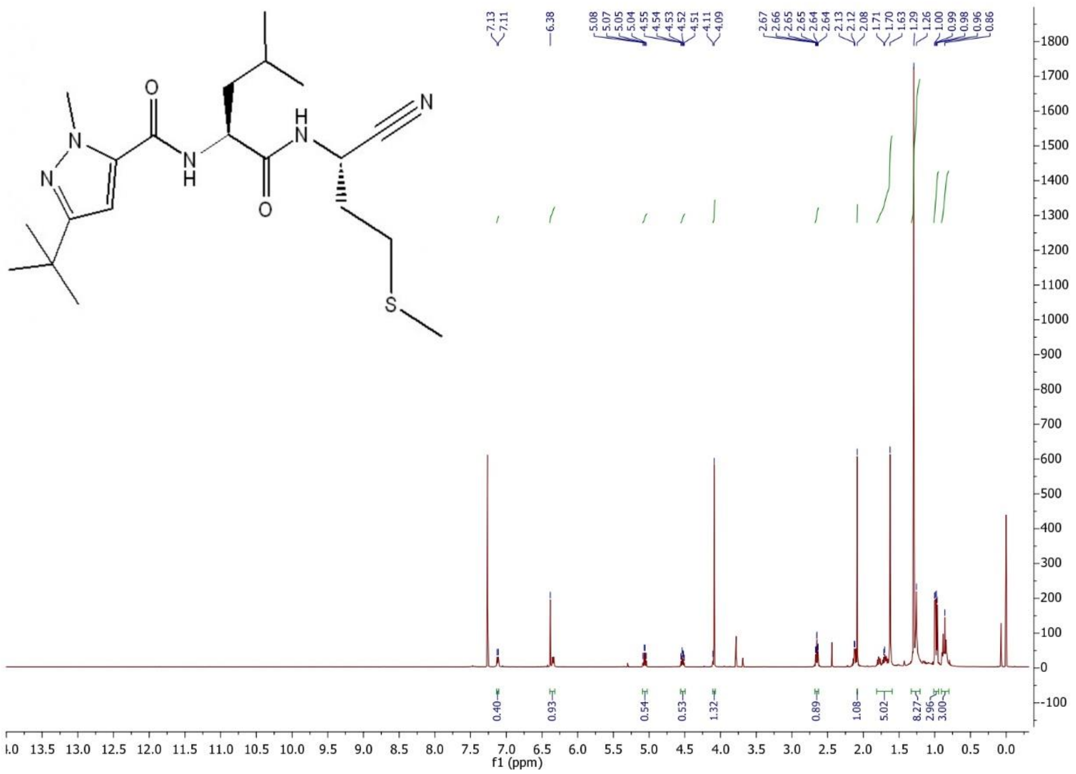
f1 (ppm)

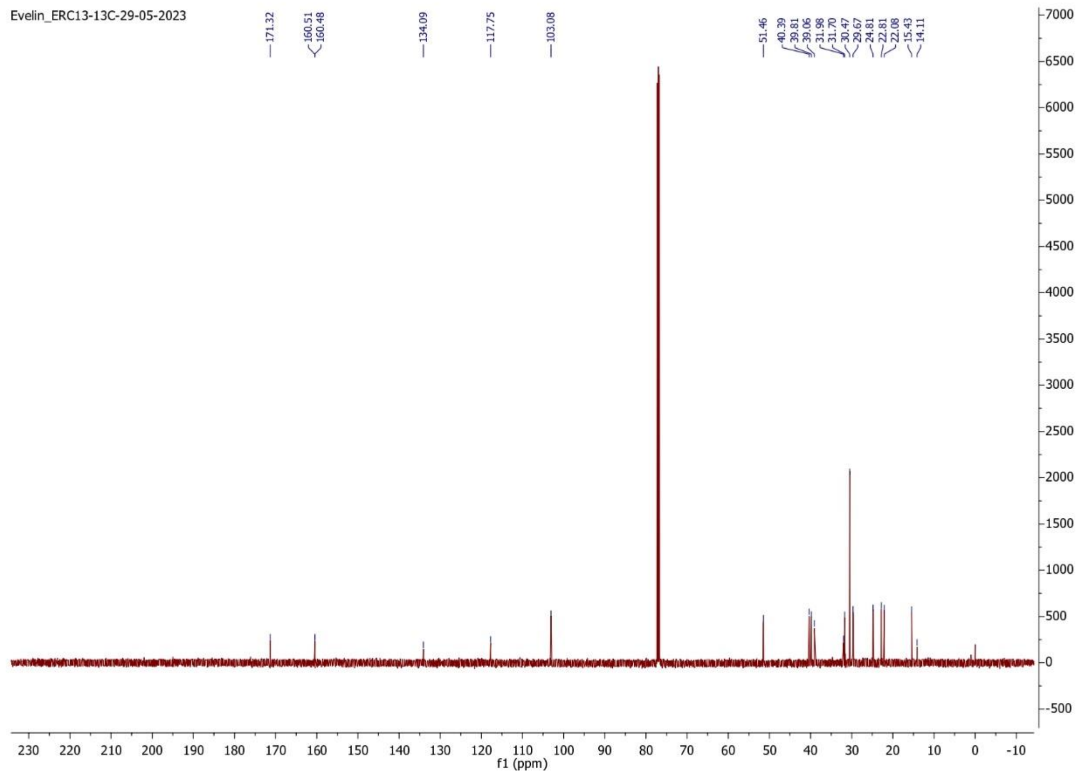
Evelin_ERC13-13C-29-05-2023
171.32
160.51
160.48
134.09
117.75
103.08
51.46
40.39
39.81
39.06
31.98
31.70
30.47
29.67
24.81
22.81
22.08
15.43
14.11
f1 (ppm)

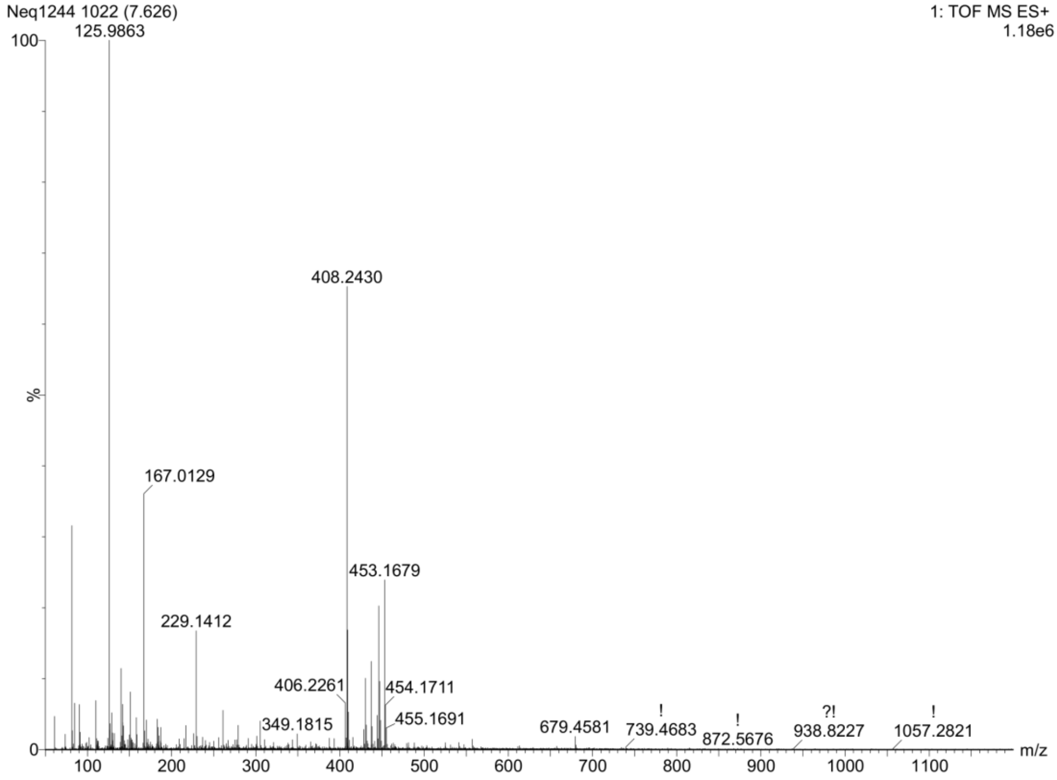
Neq1244 1022 (7.626)
1: TOF MS ES+
1.18e6
125.9863
408.2430
167.0129
453.1679
229.1412
406.2261
454.1711
349.1815
455.1691
679.4581
739.4683
872.5676
938.8227
1057.2821
%
m/z

Neq1250

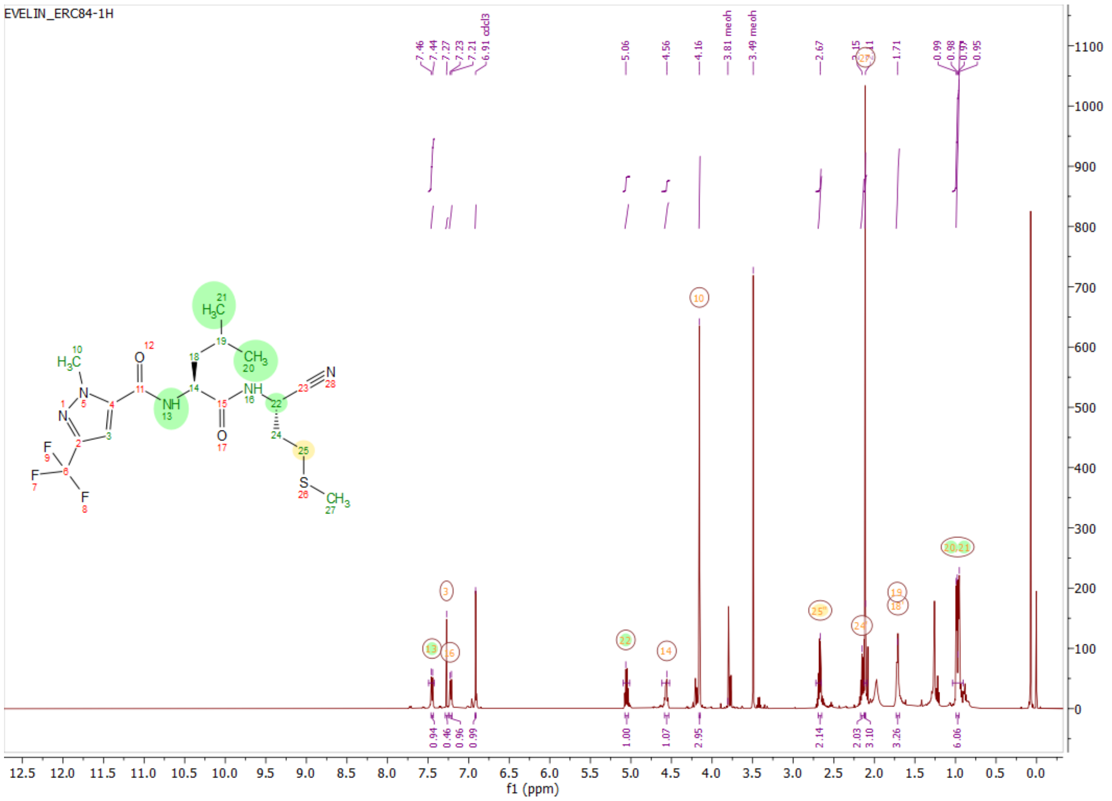

EVELIN_ERC84-1H
f1 (ppm)

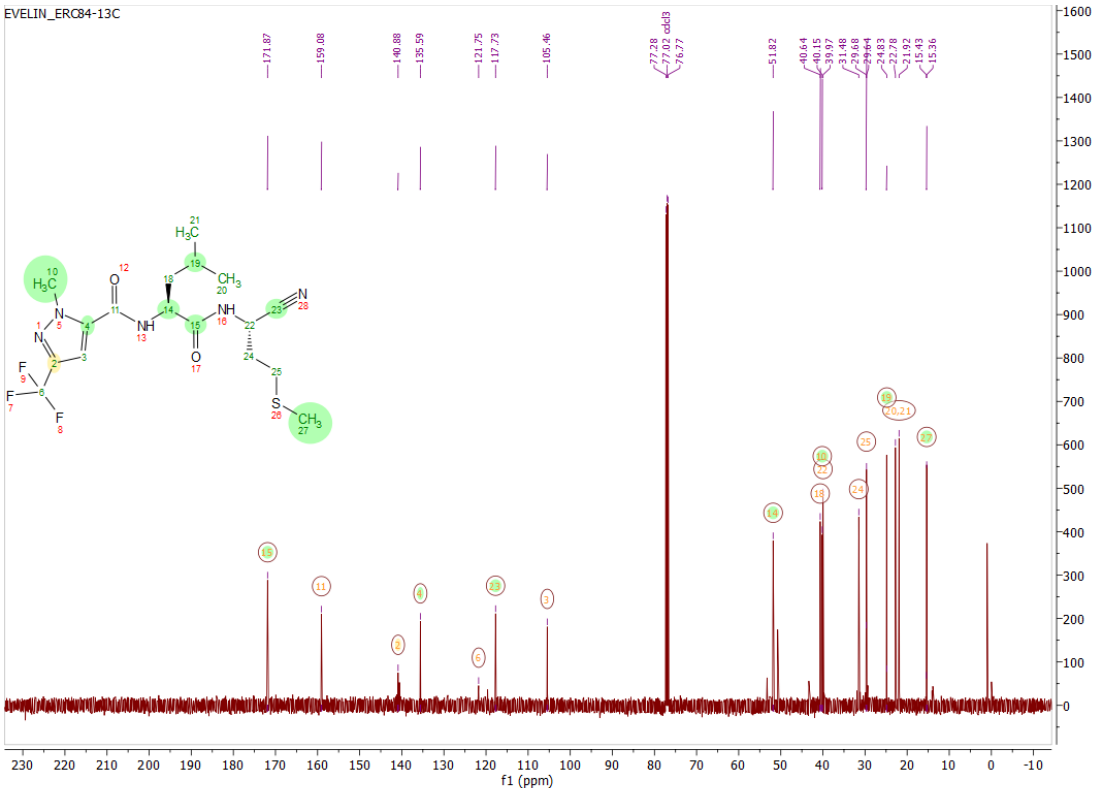

EVELIN_ERC84-13C
f1 (ppm)

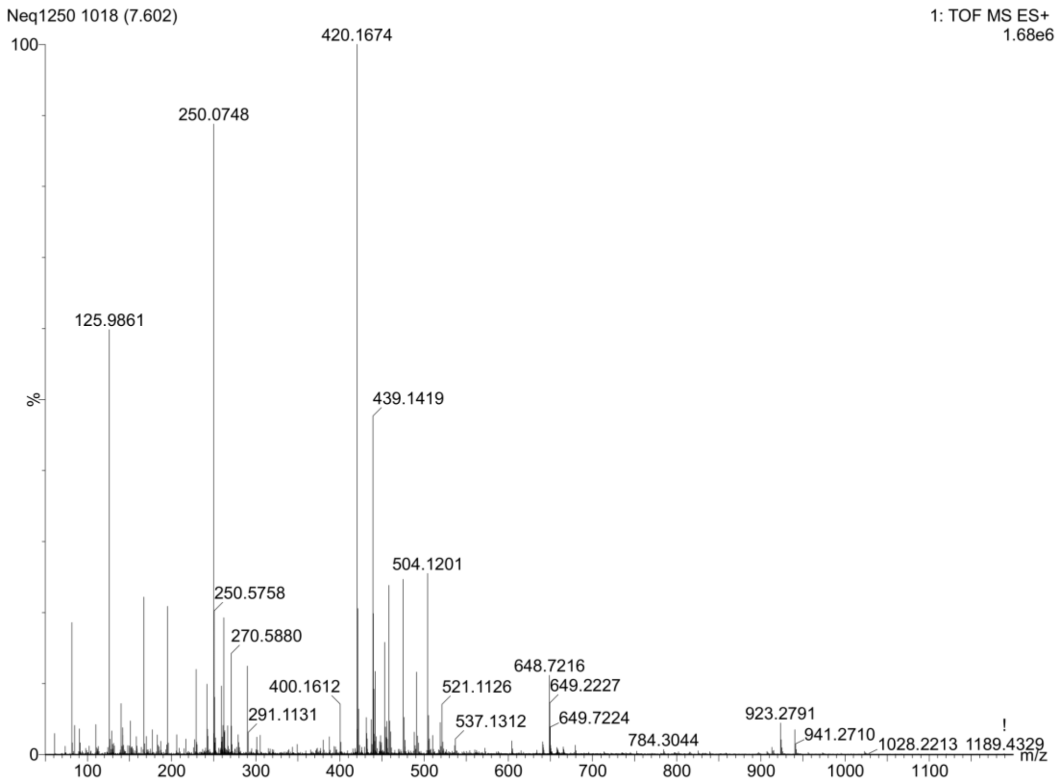

Isothermal Titration Calorimetry

Thermodynamic fingerprints of all compounds in the analysis.

Code	pK_d	ΔG	ΔH	–TΔS
Neq0414	7.3	-10.0	-13.1	3.1
Neq0533	7.2	-9.8	-12.2	2.4
Neq0568	7.4	-10.1	-10.7	0.5
Neq0569	7.6	-10.4	-15.7	5.3
Neq0940.1	7.5	-10.2	-10.7	0.5
Neq0942	7.2	-9.9	-10.8	1.0
Neq0954	7.0	-9.5	-8.1	-1.4
Neq1119	7.6	-9.8	-12.2	2.4
Neq1148	6.9	-8.8	-10.9	2.1
Neq1171	7.9	-9.1	-5.5	-3.6
Neq1182	7.2	-9.6	-10.0	0.4
Neq1244	8.0	-10.4	-17.2	6.9

Codes and structures

NeqID,Smiles

Neq0414,CN1N=C(C=C1C(=O)N[C@@H](CC1=CC=CC=C1)C(=O)NCC#N)C(C)(C)C

Neq0533,CN1N=C(C=C1C(=O)N[C@@H](CC1=CC=CC=C1)C(=O)NC1(CC1)C#N)C(C)(C)C

Neq0568,CC(C)C[C@H](NC(=O)C1=CC(=NN1C)C(C)(C)C)C(=O)NCC#N

Neq0569,CC(C)C[C@H](NC(=O)C1=CC(=NN1C)C(C)(C)C)C(=O)NC1(CC1)C#N

Neq0940.1,CC(C)C[C@H](NC(=O)[C@H](CC1=CC=CC=C1)NC(=O)C1=CC(=NN1C)C(C)(C)C)C#N

Neq0942,CC(C)C[C@H](NC(=O)C1=CC(=NN1C)C(C)(C)C)C(=O)N[C@@H](C#N)[C@@H](C)O

Neq0954,CN1N=C(C=C1C(=O)N[C@@H](CC1=CC=CC=C1)C(=O)N[C@@H](CC1=CN=CC=C1)C#N)C(C)(C)C

Neq1119,CSCC[C@@H](C#N)NC([C@@H](NC(C1=CC(C(C)(C)C)=NN1C)=O)CC2=CC=CC=C2)=O

Neq1122,COC(=O)[C@H](C[C@@H]1CNC(=O)C1)NC(=O)[C@H](CC1=CC=CC=C1)NC(=O)C1=CC(=NN1C)C(C)(C)C

Neq1133,CC(C)C[C@H](NC(=O)C1=CC(=NN1C)C(C)(C)C)C(=O)N[C@@H](C[C@@H]1CCNC1=O)C=NO

Neq1134,CON=C[C@H](C[C@@H]1CCNC1=O)NC(=O)[C@H](CC(C)C)NC(=O)C1=CC(=NN1C)C(C)(C)C

Neq1137,COC(=O)[C@H](CCSC)NC(=O)[C@H](CC1=CC=CC=C1)NC(=O)C1=CC(=NN1C)C(C)(C)C

Neq1148,CSCC[C@H](NC(=O)[C@H](CC1=CC=CC=C1)NC(=O)C1=CC(=NN1C)C(F)(F)F)C#N

Neq1171,CC(C)C[C@H](NC(=O)C1=CC(=NN1C)C(C)(C)C)C(=O)N[C@@H](C[C@@H]1CCNC1=O)C#N

Neq1172,CC(C)C[C@H](NC(=O)C1=CC(=NN1C)C(F)(F)F)C(=O)N[C@@H](C[C@@H]1CCNC1=O)C#N

Neq1182,O=C(C1=CC(C(C)(C)C)=NN1C)N[C@H](C(N[C@H](C#N)C[C@@H]2CCNC2=O)=O)CC3=CC=CC=C3

Neq1244,CSCC[C@@H](C#N)NC([C@@H](NC(C1=CC(C(C)(C)C)=NN1C)=O)CC(C)C)=O

Neq1250,CSCC[C@@H](C#N)NC([C@@H](NC(C1=CC(C(F)(F)F)=NN1C)=O)CC(C)C)=O
